# The Impact of Nonpharmacological Interventions on Opioid Use for Chronic Noncancer Pain: A Scoping Review

**DOI:** 10.3390/ijerph21060794

**Published:** 2024-06-18

**Authors:** Zhanette Coffee, Kevin Cheng, Maribeth Slebodnik, Kimberly Mulligan, Chong Ho Yu, Todd W. Vanderah, Judith S. Gordon

**Affiliations:** 1 College of Nursing, University of Arizona, Tucson, AZ 85721, USA; judithg@arizona.edu; 2 College of Medicine, University of Arizona, Tucson, AZ 85721, USA; kevincheng1@arizona.edu; 3 Health Sciences Library, University of Arizona, Tucson, AZ 85721, USA; slebodnik@arizona.edu; 4 Veterans Health Administration, Central California, Fresno, CA 93706, USA; 5 Department of Mathematics, Hawaii Pacific University, Honolulu, HI 96813, USA; chonghoyu@gmail.com; 6 Department of Pharmacology, Comprehensive Center for Pain and Addiction, University of Arizona, Tucson, AZ 85721, USA; vanderah@arizona.edu

**Keywords:** chronic noncancer pain, opioid use, nonpharmacological interventions, review

## Abstract

Despite the lack of evidence, opioids are still routinely used as a solution to long-term management for chronic noncancer pain (CNCP). Given the significant risks associated with long-term opioid use, including the increased number of unregulated opioid pills at large in the opioid ecosystem, opioid cessation or reduction may be the desired goal of the patient and clinician. Viable nonpharmacological interventions (NPIs) to complement and/or replace opioids for CNCP are needed. Comprehensive reviews that address the impact of NPIs to help adults with CNCP reduce opioid use safely are lacking. We conducted a literature search in PubMed, CINAHL, Embase, PsycINFO, and Scopus for studies published in English. The initial search was conducted in April 2021, and updated in January 2024. The literature search yielded 19,190 relevant articles. Thirty-nine studies met the eligibility criteria and underwent data extraction. Of these, nineteen (49%) were randomized controlled trials, eighteen (46%) were observational studies, and two (5%) were secondary analyses. Among adults with CNCP who use opioids for pain management, studies on mindfulness, yoga, educational programs, certain devices or digital technology, chiropractic, and combination NPIs suggest that they might be an effective approach for reducing both pain intensity and opioid use, but other NPIs did not show a significant effect (e.g., hypnosis, virtual reality). This review revealed there is a small to moderate body of literature demonstrating that some NPIs might be an effective and safe approach for reducing pain and opioid use, concurrently.

## 1. Introduction

Chronic noncancer pain (CNCP) is defined as nonmalignant pain that persists past the anticipated healing times or for greater than 3 months and affects up to 20% of the world’s population [1]. CNCP is a biopsychological disorder, involving a complex interplay of psychological, social, and biological factors that synergistically contribute to individualized pain experiences, and most often requires a personalized multimodal approach to management [2,3]. Despite clinical guidelines recommending limiting opioids and promoting the use of nonpharmacological interventions (NPIs) as first line therapies for treating CNCP [4,5,6], opioid prescriptions are still the most common treatment used for CNCP [4,7]. The U.S. has less than 5% of the world’s population but consumed more than 99% of the world’s hydrocodone and 80% of the world’s oxycodone in 2009, which is more opioid consumption per capita than any other country in the world [8,9]. In individuals using opioid treatment for CNCP, the likelihood of rehabilitation is four times lower than nonopioid alternatives [10]. The use of long-term opioids to treat benign, noncancer pain is not an evidence-based approach [11,12] and in many CNCP conditions, opioid use increases medication tolerance and reliance, and decreases function [13]. Reliance on opioid medications to treat CNCP has led to an epidemic of opioid-related disability, misuse, dependence, addiction, and mortality [14].

Opioids are a controlled (scheduled by the Drug Enforcement Administration) class of analgesic medications that are either opiates (derived from opium poppy) or opioid-like (semi-synthetic or synthetic medications that mimic the mechanism of action of opiates) [5]. Opioid receptors have been found in diverse non-neuronal human tissues [15]. Prescription opioids have wide-ranging side effects including constipation, sedation, nausea [16], the suppression of the immune system due to direct interactions between opioids and immune cells, such as T cells and B cells [17], hypogonadism [18], an increased risk of osteoporosis and fracture due to the disruption of the production and release of estrogen, testosterone, and cortisol [19], slowed wound healing due to impairing blood flow and delaying cell proliferation [20,21], the exacerbation of central and obstructive sleep apnea [22,23], arrhythmogenesis and myocardial events [24], an increased risk of depression caused by neurotransmitter imbalances and hormonal changes [25], psychomotor retardation contributing to falls [26,27], and the worsening of pain due to neurogenic inflammation and hyperalgesia [28]. Abruptly tapering opioid prescriptions without an effective, alternative treatment has exacerbated the problem with unpleasant side effects, increased pain intensity, and a shift from prescription opioid use to illicit substance use disorder and mental health crisis, including suicide attempts [29]. In light of the current opioid crisis and an increasing prevalence of CNCP, there is an urgent need to identify safer multimodal therapeutic approaches that effectively manage the perception of pain, reduce opioid use, and concurrently target the pain-generating source [1,10,30].

Nonpharmacological interventions (NPIs) are broad ranging and can be grouped as oriented towards physical, psychological, or clinical process aspects of care [31]. NPIs may include acupuncture or neurostimulation, mindfulness-based stress reduction, cognitive behavioral therapy, hypnosis, spirituality, tai chi/qigong, yoga, massage therapy, multidisciplinary rehabilitation, self-management programs, educational programs, devices, digital health technology, and manipulative therapies [4,32]. NPIs may address emotion dysregulation, pain, and reward-processing deficits that often drive opioid use, tolerance, and dependence [33]. NPIs can be complementary to traditional medical treatments for CNCP to reduce the need for higher dosages and to minimize possible side effects associated with medications.

There is minimal scientific exploration of the effectiveness, clinical impact, safety, and implementation of NPIs to reduce opioid consumption and pain-related symptoms in CNCP individuals [34]. A comprehensive evaluation of NPIs for CNCP individuals who use opioids for pain management will provide information for clinicians and patients to evaluate alternative evidence-based interventions to manage pain-related symptoms and safely reduce opioid consumption. For this reason, we reviewed the literature on the impact of NPIs on opioid use among adults with CNCP.

The following research questions guided the review:

(1) What are the characteristics (e.g., type, duration, frequency, setting) of NPIs that are used for adults with CNCP?
(2) What impact does the use of NPIs have on opioid use and pain-related outcomes in adults with CNCP?

## 2. Methods

This scoping review followed the extension of the Preferred Reporting Items for Systematic Reviews and Meta-Analysis framework for scoping reviews (PRISMA-ScR)—the checklist can be found in the supplemental material (Appendix A) [35]. We registered our scoping review protocol in Open Science Framework (https://osf.io/j7kwy)—accessed on 11 April 2024. The detailed search strategy for this scoping review can be found in the supplemental material (Appendix B). The Mixed Methods Appraisal Tool (MMAT) [36] was used to assess the quality of the included studies based on each study’s reporting, external validity, internal validity, and power. The authors contributed to the discussion regarding the overall quality and risk of bias in the included studies. If data or analyses were not clear or available, it was reflected accordingly using the MMAT.

### 2.1. Inclusion and Exclusion Criteria

Studies with the following attributes were included: (1) randomized clinical trials and/or mixed methods study designs; (2) quantitative studies on nonpharmacological treatments; (3) nonmalignant and/or noncancer chronic pain treatment; (4) adult study participants; (5) English, full-length articles; and (6) quantitative studies that measure opioid use. Studies with the following attributes were excluded: (1) Studies on cannabis and/or cannabinoids; (2) animal/basic science studies; (3) studies in which there were no chronic pain diagnoses; (4) treatment for cancer pain, palliative care pain, pain at end-of-life, sickle cell pain, HIV pain, acute pain, postoperative pain, or labor pain; (5) conference proceedings or abstracts; (6) pediatric participants; (7) non-English articles; (8) studies that used invasive interventions for chronic pain (e.g., spinal cord stimulators, trigger point injections, percutaneous peripheral nerve stimulator, surgical interventions); (9) studies that did not exclusively measure opioid use; (10) systematic reviews or other review types, and (11) studies that used pharmaceutical interventions.

It is worth noting that although cannabis or cannabinoids may be used as an integrative approach for pain, we excluded cannabis or cannabinoids as the literature does not support the effectiveness of this approach for reducing both pain and opioid use. Concurrently, as revealed by a systematic review on the efficacy of integrative medicine approaches to reduce prescribed opioid use for chronic pain, participants who used cannabis had a greater pain severity score, greater pain interference score, lower pain self-efficacy scores, and greater anxiety severity scores [34]. Additionally, the literature shows that recreational and medical cannabis legalization were not associated with significant decreases in opioid prescriptions and fatal overdoses [37].

The NPIs selected for this review were informed by guidelines recommended by several health care agencies and medical societies: Academic Consortium for Integrative Medicine and Health, Veterans Health Association, Center for Disease Control and Prevention, American College of Physicians, and Agency for Healthcare Research and Quality. The NPI selected terms were as follows: cognitive behavioral therapy, chiropractic, neurostimulation, biofeedback, acupuncture, acupressure, tai chi, massage, yoga, qigong, meditation, guided imagery, music therapy, art therapy, therapeutic touch, complementary and alternative therapies, and biopsychosocial. It is worth noting that there is a unique spectrum of opioid use—there is a difference between long-term opioid use (taking prescribed opioid medications for greater than 3 months) and opioid use disorder (per the Diagnostic and Statistical Manual or Mental Disorders, Fifth Edition) or opioid addiction (chronic use of prescribed or illicit opioids that include drug-seeking behaviors that result in clinically significant distress or impairment to life). Not all CNCP individuals that use opioids as prescribed to treat pain symptoms will experience aberrant drug-related behaviors (e.g., overuse, abuse, misuse, addiction). However, since nearly 30% of individuals who have had opioids prescribed long-term exhibit such aberrant behaviors [38], we included these opioid terms: opioid-related disorders, opioid addiction, opiate abuse, opioid abuse, opiate dependence, analgesics, opioids.

### 2.2. Search Strategy

A comprehensive electronic search was performed in April 2021 and updated in January 2024. The results were deduplicated in EndNote 20 software. The literature search was designed using subject headings and free text terms and performed by a medical librarian (MS) in the following databases: PubMed, CINAHL, Embase, PsycINFO, and Scopus. Filters were used to limit to English language articles and human research subjects. No restrictions on publication date were imposed.

### 2.3. Data Screening

Two independent reviewers screened titles, abstracts, and full texts for inclusion (ZC, KC, KM, and MS) using predetermined criteria. The results were screened using Microsoft Excel for the initial title abstract screening, and DistillerSR software (versions 2.1–2.4) for the final screening steps. The team members performed a norming exercise prior to screening to ensure inter-rater consistency. Any initial discrepancies were resolved by discussion until consensus about inclusion or exclusion was achieved. The reference lists of study reports included in the review were carefully examined for additional citations.

### 2.4. Data Extraction

Three reviewers (ZC, KC, KM) extracted data independently in a standardized manner using Microsoft Excel and Distiller SR. In the data extraction phase, the following data were collected: study design, sample size, mean subject age, subject age range, military status, sex, setting, study duration, intervention type, details of intervention, data collection protocol, descriptive statistics, inferential statistics, pain type/location, pain scale(s), measure of opioid use, other measures/outcomes, study results, study limitations, and additional notes.

### 2.5. Data Analysis

All included studies were analyzed qualitatively. NPIs were classified into specific types according to their characteristics. A reported result with a *p*-value less than 0.05 was considered to be statistically significant.

## 3. Results

**Figure 1:**
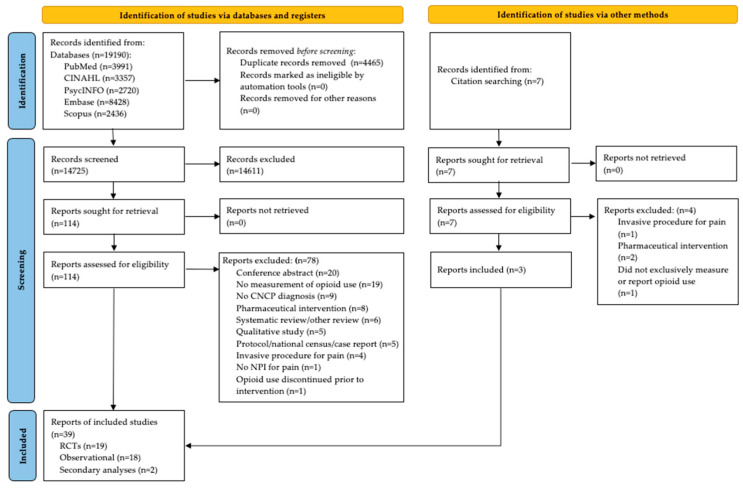
Preferred reporting items of scoping reviews flow chart.

The search yielded 19,190 potential articles. All potential articles were screened using MS Excel for the initial title abstract screening, and DistillerSR software (versions 2.1–2.4) for the final screening steps. After removing duplicates, completing screening, and carefully reviewing the reference lists of relevant articles, a total of 39 articles met the eligibility criteria and underwent data extraction (Figure 1 below).

As shown in Figure 1, the 39 studies used a broad range of research designs; nineteen randomized controlled trials (RCTs) [2,7,29,30,38,39,40,41,42,43,44,45,46,47,48,49,50,51,52], eighteen non-randomized observational studies (e.g., prospective and retrospective cohort studies, case studies, quasi-experimental studies) [1,3,5,10,14,53,54,55,56,57,58,59,60,61,62,63,64,65], and two secondary analyses of RCTs [66,67] met the inclusion criteria. The final 39 articles were tabled and analyzed according to the level of hierarchy (Table 1 below and supplementary material Table S1: Characteristics of Included Studies).

### 3.1. Characteristics of Included Studies

A total of 28,346 participants with CNCP enrolled in the 39 studies. Thirty-three [85%, 33/39] studies reported the mean age (54.5 years old) [1,2,3,7,14,29,38,40,41,42,44,45,46,47,48,49,50,51,52,54,55,56,57,58,59,60,61,62,64,65,66,67] and six [15%, 6/39] studies did not report mean age [5,10,30,39,53,63]. Thirty-nine [100%, 39/39] studies provided the biological sex of the study participants and the percentage of females ranged from 9.2% [50] to 100% [66]. Twenty-eight [72%, 28/39] studies reported race and ethnicity characteristics. Only four studies [10%, 4/39] included a sample of >50% underrepresented populations [44,51,65,66]. Miller-Matero et al. (2022) had the highest percentage of Black (88.3%) participants, and Sandhu et al. (2023) had the highest percentage of White (96.2%) participants, respectively, as shown in supplementary material Table S1: Characteristics of Included Studies. Nine studies [23%, 9/39] included a sample of veteran or active military personnel participants [10,14,38,41,50,51,56,63,65]. Three studies [8%, 3/39] tested NPIs for CNCP patients with problematic opioid use or opioid misuse [14,43,61]. The types of CNCP reported were as follows: generalized [2,42,54,59,63,64], back or chronic low back (cLBP) [1,2,3,5,7,39,40,41,42,43,45,46,48,53,54,57,59,63,64], fibromyalgia [3,5,7,38,42,43,45,46,49,54,55,59,60,63,64,67], chronic musculoskeletal [52,66], spinal cord injury or spine-related [39,47,56,58], neuropathic or neuropathy [3,5,38,42,43,62], multiple sclerosis [39], acquired amputation [39], muscular dystrophy [39], complex regional pain syndrome [3], joints or arthritis [3,5,38,41,43,45,46,53,55], pelvic [3,42,43], migraine or headaches [3,38,42,43,46,53,55,56,63,64], cervical [38,43,58], sacral [58], neck or shoulders [1,3,7,41,46,53,55,59,63], abdominal-related [42,43,53,56,59], musculoskeletal chest [1,42,63], orofacial or jaw [42,53], carpal tunnel [53], hip [1,53], extremity [1,7,38,42,56,58,63], and other or unspecified [3,5,7,10,14,29,38,41,42,43,45,46,53,54,59,61,63,64,65]. Thirty-seven [95%, 37/39] studies were conducted in outpatient settings, one study [2.5%, 1/39] was conducted in a residential setting [57], and one study [2.5%, 1/39] was conducted in both inpatient and outpatient settings [3]. The studies included in this review reported a duration of NPI treatment from 10 days to 22 months, with the most common duration being eight weeks. Seventeen [44%, 17/39] studies clearly reported adverse events ranging from none [1,39,43,50,53], minor (related to participation) [29,30,40,46,49,51,62], and serious (unrelated to participation) [2,42,44,47,48,49]. The publication years ranged from 2007 to 2024.

**Table 1:** Characteristics of Included Studies.

First Author, Year	Design	N	NPI Type	Duration	Pain and Opioid Use Measures	Pain Intensity and Opioid Use Results
Garcia, 2021	RCT	179	Device	56 days	Pain: Defense and Veterans Pain Rating Scale Opioid use: Self-reported and converted to morphine milligram equivalent (MME)	Pain: Pain intensity reduced by an average of 42.8% for the virtual reality (EaseVRx) group and 25% for the sham virtual reality group. Opioid use: Did not reach statistical significance for either group.
Jensen, 2020	RCT	173	Hypnosis	4 sessions	Pain: Numeric Rating Scale Opioid use: Self-reported and converted to MME	Pain: No statistically significant between-group differences on omnibus test for pain intensity. On average, pain intensity reduced between pre- vs. post-treatment for all groups. Opioid use: No changes in opioid use were found.
Zheng, 2019	RCT	108	Acupuncture	12 wks	Pain: Visual Analogue Scale Opioid use: Self-reported and converted to MME	Pain: No group differences were found in pain intensity. No changes in pain intensity were found over time. Opioid use: Opioid use reduced by 20.5% (*p* < 0.05) and 13.7% (*p* < 0.01) in the two acupuncture groups and by 4.5% in the education group post-treatment, but without any group differences. For follow-up, the education group had a 47% decrease in opioid use after a course of electroacupuncture.
Garland, 2022	RCT	250	Mindfulness	8 wks	Pain: Brief Pain Inventory Opioid use: Urine toxicologic screening, Self-reported and converted to MME	Pain: MORE showed greater reductions in pain severity (between-group effect: 0.49; 95% CI, 0.17–0.81; *p* = 0.003) than the control group. Opioid use: MORE reduced the opioid use more than the control group (between-group effect: 0.15 log mg; 95% CI, 0.03–0.27 log mg; *p* = 0.009). At 9-month follow-up, 22 of 62 participants (35.5%) in MORE group reduced opioid use by at least 50%, compared to 11 of 69 participants (15.9%) in the control group (*p* = 0.009). At 9-months, 36 of 80 participants (45.0%) in MORE were no longer misusing opioids compared with 19 of 78 participants (24.4%) in the control group.
Hudak, 2021	RCT	62 *	Mindfulness	8 wks	Pain: NA Opioid use: Self-reported and converted to MME	Pain: NA Opioid use: Participants in MORE showed greater reduction in opioid use over time than the control group.
Wilson, 2023	RCT	402	Educational Program	8 wks	Pain: Brief Pain Inventory Opioid use: Opioid prescription information was collected from the participants medical record and converted to MME	Pain: 24 (14.5%) of 166 E-Health participants achieved a >2 point decrease in pain intensity compared to 13 (6.8%) of 192 TAU participants (odds ratio, 2.4 [95% CL, 1.2–4.9]; *p* = 0.02). Opioid use: 105 (53.6%) of 196 E-Health participants achieved a >15% reduction in opioid use compared with 85 (42.3%) of 201 TAU participants (odds ratio, 1.6 [95% CL, 1.1–2.3]; *p* = 0.02).
Garland, 2024	RCT	230 *	Mindfulness	8 wks	Pain: Brief Pain Inventory Opioid use: Urine drug screens, opioid prescription information was collected from the participants medical record and converted to MME	Pain: MORE showed significantly greater reduction in pain outcomes than the control group (*p* = 0.025). Opioid use: MORE reduced opioid dose significantly compared to control group (B = 0.65, 95% CI = 0.07–1.23, *p* = 0.029); 20.7% reduction in mean opioid use (18.88 mg, SD = 8.40 mg) for MORE compared to 3.9% reduction (3.19 mg, SD = 4.38 mg) for control group. MORE showed significantly greater reduction in opioid dose than control group (*p* = 0.025).
DeBar, 2022	RCT	850	CBT	12 wks	Pain: Pain Intensity and Interference with Enjoyment of Life, General Activity, and Sleep Opioid use: Self-reported and converted to MME per 90-day period	Pain: CBT had larger reductions in pain outcomes at 12-month follow-up compared to usual care (difference, −0.434 point [95% CI, 0.690 to −0.178 point]) and post-treatment (difference, −0.565 point [CI, −0.796 to −0.333 point]). Opioid use: No differences were seen in opioid use at post-treatment (difference, −2.260 points [CI, −5.509 to 0.989 points]) or at 12-month follow-up (difference, −1.969 points [CI, −6.765 to 2.827 points]).
Gardiner, 2019	RCT	159	Combined	21 wks	Pain: Brief Pain Inventory Opioid use: Self-reported	Pain: No differences in pain outcomes at any time point. Opioid use: At 21 weeks, the IMGV group reported greater reduction in pain medications use (Odds Ratio: 0.42, CI: 0.18–0.98) compared to controls.
Wartko, 2023	RCT	153	CBT	18 sessions/ 1 year	Pain: Pain, Enjoyment of life, and General activity Opioid use: Self-reported and converted to MME	Pain: No significant differences between intervention and usual care for pain outcomes were found (0.0 [95% CI: −0.5, 0.5], *p* = 0.985). Opioid use: No significant differences between intervention and usual care for opioid use were found (adjusted mean difference: −2.3 MME; 95% CI: −10.6, 5.9; *p* = 0.578).
Groessl, 2017	RCT	150 *	Yoga	12 wks	Pain: Brief Pain Inventory Opioid use: Self-report and verified using medical records.	Pain: Differences observed at all three time points (*p* = 0.001 for 6 weeks, 0.005 for 12 weeks, 0.013 for 6 months), with larger reductions in pain intensity for yoga participants. Opioid use: Significant reduction from 20% to 11% at 12 weeks (*p* = 0.007) and 8% after 6 months (*p* < 0.001).
Roseen, 2022	RCT	120 *	Yoga	12 wks	Pain: Defense and Veterans Pain Rating Scale Opioid use: Self-reported	Pain: No significant in-between differences were observed for pain. Opioid use: No significant in-between differences were observed for opioid use. Post-treatment, fewer yoga than education participants reported pain medication use (55% vs. 67%, OR = 0.56, 95% CI: 0.26–1.24, *p* = 0.15).
Sandhu, 2023	RCT	608	Educational Program	3 days and 12 months maintenance	Pain: Patient-Reported Outcomes Measurement Information System Opioid use: Self-reported, with a participant report verified in a telephone call from a member of the study team and converted to MME	Pain: No significant between-group differences in pain intensity. Opioid use: At 12 months, 65 of 225 participants (29%) achieved opioid cessation in the intervention group and 15 of 208 participants (7%) achieved opioid cessation in the usual care group (odds ratio, 5.55 [95% CI, 2.80 to 10.99].
Does, 2024	RCT	376	Educational Program	4 sessions	Pain: Patient-Reported Outcomes Measurement Information System Opioid use: Pharmacy dispensation data from the medical record and converted to MME for the 6-month period.	Pain: No significant between-group differences in pain intensity. Opioid use: A small but not significant decrease in opioid use was found in both groups over the study period. At 12 months, intervention group demonstrated greater medication use (OR = 2.72; 95% CI 1.61–4.58).
Naylor, 2010	RCT	51	Digital Technology	4 months	Pain: Short form of the McGill Pain Questionnaire, the Pain Symptoms Subscale from the Treatment Outcomes in Pain Survey Opioid use: Self-reported	Pain: TIVR showed significant improvement at 8-month follow-up for pain scores (*p* < 0.0001), compared to the control group. Opioid use: Opioid use reduced in the TIVR group in both follow-ups: 4- and 8-months post CBT. At 8-month follow-up, 21% of the TIVR participants stopped using opioids. There was significant between group differences in opioid use at 8-month follow-up (*p* = 0.004).
Nielssen, 2019	RCT	50	Educational Program	8 wks	Pain: Roland–Morris Disability Questionnaire, Wisconsin Brief Pain Questionnaire Opioid use: Self-reported and converted to MME	Pain: Significantly larger reduction in pain outcomes with the intervention compared to the control group. Opioid use: Significant reduction in opioid use compared to control group.
Day, 2019	RCT	69	Combined	8 wks	Pain: Numeric Rating Scale Opioid use: Self-reported opioid use in the past week	Pain: Post-treatment, the intent-to-treat group showed significant improvements for pain intensity (*p* < 0.001), with no significant between group differences. Opioid use: For the intent-to-treat group, there was no significant difference (*p* = 0.549) in opioid use between pre-treatment (48%) and post-treatment (43%). Opioid use decreased significantly (*p* = 0.012) from pre-treatment (49%) to 3-month follow-up (28%), but opioid use at post-treatment (40%) and 6-month follow-up (33%) were not significantly reduced (*p* = 0.289) than at pre-treatment.
Spangeus, 2023	RCT	21	Educational Program	10 wks	Pain: Numeric Pain Scale Opioid use: Self-reported opioid use	Pain: Significant improvements post-treatment on pain outcomes were found. Opioid use: Significant reduction in opioid use (25%) at baseline and (14%) at post-treatment were found.
Nelli, 2023	OB	45	Device	2 wks	Pain: Numeric Scale Opioid use: Self-reported and converted to MME	Pain: The reduction in pain scores was 67%, 50%, and 45% for the green, blue, and clear glasses groups (*p* = 0.56). No significant differences in pain score reduction between groups was found. Opioid use: Greater than 10% reduction in opioid use was achieved and found 33%, 11%, and 8% of the green, blue, and clear eyeglasses groups (*p* = 0.23).
Moffat, 2023	OB	13,968 *	Combined	22 months	Pain: NA Opioid use: Identified using the Australian Pharmaceutical Benefits Scheme item number and converted to MME	Pain: NA. Opioid use: Calculated change in predicted trends with and without the intervention 25,387 (95% CI 24,676, 26,131).
Zeliadt, 2022	OB	4869 *	Combined	18 months	Pain: NA Opioid use: Extracted from VA’s pharmacy managerial cost accounting national data extract and converted to MME.	Pain: NA. Opioid use: Opioid use decreased by −12% in one year among veterans who began CIH compared to similar veterans who used conventional care; −4.4% among veterans who used only Whole Health services compared to conventional care, and −8.5% among veterans who used both CIH combined with Whole Health services compared to conventional care.
Huffman, 2019	OB	1681	Combined	4 wks	Pain: Numeric Rating Scale Opioid use: Self-reported	Pain: Pain on discharge, and at 6 months and 12 months was significantly lower compared to on admission (*p* < 0.05). Opioid use: There were significantly fewer patients using opioids *p* < 0.05) post-treatment. At 6-month follow-up, 76.3% maintained opioid cessation, 14.6% resumed opioid use, 5.8% remained continued to use opioids, and 3.4% discontinued opioid use. At 12-month follow-up, 14.6% maintained opioid cessation, 5.8% resumed opioids, 3.4% continued to use opioids, and 76.3% discontinued opioid use.
Townsend, 2008	OB	373	Combined	3 wks	Pain: Multidimensional Pain Inventory Opioid use: Verified using medical records and converted to MME	Pain: Significant improvement was found in pain outcomes post- treatment (*p* < 0.001) and six months post-treatment (*p* < 0.001). Opioid use: At discharge, 176 (92.6%) of the opioid group had completed the taper of opioids (*x*^2^ = 20.57; df = 1, *p* < 0.001).
Ward, 2022	OB	237 *	Combined	10 wks	Pain: Pain Numeric Scale Opioid use: Number of days with prescription opioids determined from VA pharmacy data	Pain: No significant improvement to pain scores noted. Opioid use: No significant differences in percentage of opioid use found one year pre-post treatment for both EVP engaged and not engaged participants.
Van Der Merwe, 2021	OB	164 *	Combined	10 days	Pain: Brief Pain Inventory Opioid use: Self-reported	Pain: Significant improvement with treatment (*p* < 0.001). Opioid use: Approximately, 25% ceased opioid use and 17% had reduced opioid use post-treatment.
Hooten, 2007	OB	159	Combined	3 wks	Pain: Multidimensional Pain Inventory Opioid use: Medical chart review	Pain: Significant improvement with program treatment (*p* < 0.001). Opioid use: Compared with admission, opioid use at post-treatment was significantly reduced (*p* < 0.001).
Davis, 2018	OB	156	Acupuncture	12 sessions/60 days	Pain: Patient-Reported Outcomes Measurement Information System Opioid use: Self-reported	Pain: Significant improvements in pain intensity (*p* < 0.01). Opioid use: Approximately 32% of patients using opioids reported reductions in use post-intervention.
Schumann, 2020	OB	134	Combined	3 wks	Pain: West Haven Yale Multidisciplinary Pain Inventory Opioid use: Self-reported and converted to MME	Pain: Significant treatment effects (*p* < 0.001) with large effect sizes were observed. Opioid use: Significant reductions (*p* < 0.01) in opioids were found post-treatment. All participants in the opioid group completed the opioid taper and discontinued use.
Gibson, 2020	OB	99 *	Combined	3 months	Pain: Brief Pain Inventory Opioid use: Self-reported	Pain: No significant change in pain severity (*p* = 0.11, *ES* = 0.16). Opioid use: At baseline, 77 participants were prescribed opioids, 6 (7%) discontinued use between baseline and follow-up.
Van Hooff, 2012	OB	85	Combined	10 days	Pain: Visual Analogue Scale Opioid use: Self-reported	Pain: No significant improvement at 1-year follow-up (*p* = 0.34). Opioids use: Minimal reduction was found, 25% of patients used opioids (15% weak opioid, 10% strong opioid) at pre-treatment, and 14% of patients used opioids (11% weak opioid, 3% strong opioid) at 2-year follow-up.
Gilliam, 2020	OB	762	Combined	15 days	Pain: West Haven Yale Multidimensional Pain Inventory Opioid use: Medical records, medicine bottles, patient report, and state prescription monitoring programs and converted to MME	Pain: Significant improvements were found for pain outcomes. Opioid use: Significant improvements were found for opioid use. At discharge, all patients (31.8%, *n* = 242) taking opioids at pre-treatment had completed the taper and discontinued opioid use.
Trinh, 2023	OB	74	Device	30 days	Pain: Brief Pain Inventory, Visual Analogue Scale Opioid use: Self-reported, compensation claimants	Pain: Significant reduction in pain post H-Wave treatment (*p* < 0.0001) Opioid use: Approximately, 49% of the patients taking opioids prior to the H-Wave device intervention subsequently reduced or stopped their usage.
Passmore, 2022	OB	62	Chiropractic	NA	Pain: Numeric Rating Scale Opioid use: Self-reported	Pain: Significant decrease in pain intensity was found. Opioid use: Significant reduction of opioid use was found (*p* = 0.012), approximately 59.0% reduction post-treatment.
Buchfuhrer, 2023	OB	20	Device	21 days	Pain: Clinician Global Impression of Improvement Opioid use: Self-reported and converted to MME	Pain: No changes to restless legs syndrome severity found. Opioid use: Approximately, 70% of participants (14/20) successfully reduced opioid use >20%, 29.9% mean opioid reduction (*SD* = 23.7%, *n* = 20) from 39.0 to 26.8 MME per day post-TOMAC treatment.
Barrett, 2021	OB	17	Combined	8 wks	Pain: Brief Pain Inventory Opioid use: Self-reported and converted to MME	Pain: No significant changes in pain severity (5.9 vs. 5.93, *p* = 0.913). Opioid use: Five participants (38.5%) reported decreasing their opioid use since baseline. Of these five, opioid use reductions were 17%, 25%, 34%, 55%, and 74%. The mean opioid use decreased from 138.17 mg (*SD* = 83.99) to 101.21 mg (*SD* = 45.71).
Matyac, 2022	OB	13	Educational Program	5 wks	Pain: Pain, Enjoyment, and General Activity Opioid use: Self-reported and converted to MME	Pain: The program was associated with decreased pain intensity. Opioid use: Although not significant, the program was associated with reduced opioid use.
Nilsen, 2010	OB	11	CBT	8 wks	Pain: Brief Pain Inventory Opioid use: Codeine (milligram) use and blood sample taken at the first session for genetic polymorphism CTP2D6	Pain: No significant changes (*p* > 0.05) were found to mid-treatment (*d* = 0.3), post-treatment (*d* = 0.4), or to follow-up (*d* = 0.4). Opioid use: A significant decrease in codeine use was found from pre- to mid-treatment (*t* = 11.4, *p* < 0.001; *d* = 2.2), pre-to post-treatment (*t* = 11.8, *p* < 0.001; *d* = 2.9), pre-treatment to follow-up (*t* = 11.7, *p* < 0.001; *d* = 2.9) and from mid- to post-treatment (*t* = 6.1, *p* < 0.001; *d* = 1.4).
McCrae, 2020	SA	113	CBT	8 wks	Pain: NA Opioid use: Self-reported	Pain: NA. Opioid use: There were no significant effects for frequency of opioid use between groups (CBT-insomnia, CBT-pain, waitlist control).
Miller-Matero, 2022	SA	60	Combined	5 sessions	Pain: Brief Pain Inventory Opioid use: EHRs verified and converted to MME	Pain: Intervention significantly reduced pain outcomes (*p* = 0.048). Opioid use: Though not significant, the intervention showed lower odds of having an opioid prescription 6 months post-intervention (*p* = 0.09, *OR* = 0.32).

Table 1 above is condensed. For full details, see supplementary material Table S1: Characteristics of Included Studies. Abbreviations: nonpharmacological interventions (NPIs), chronic noncancer pain (CNCP), cognitive behavioral therapy (CBT), cognitive therapy (CT), mindfulness meditation (MM), mindfulness-based cognitive therapy (MBCT), electronic medical record (EHR), Veterans Health Administration (VA), Biopsychosocial Integrated Pain Team (IPT), Brief Pain Inventory (BPI), randomized controlled trial (RCT), observational study (OB), secondary analysis (SA), Patient-Reported Outcomes Measurement Information System (PROMIS), interdisciplinary pain rehabilitation program (IPRP), morphine milligram equivalent (MME), West Haven Yale Multidimensional Pain Inventory (WHYMPI), Defense and Veterans Pain Rating Scale (DVPRS), Roland–Morris Disability Questionnaire (RMDQ), Pain Disability Index (PDI), Clinician Global Impression of Improvement (CGI-I), Multidimensional Pain Inventory (MPI), standard deviation (SD), tonic motor activation (TOMAC), acceptance and commitment therapy (ACT), Empower Veterans Program (EVP), complementary and integrative health therapies (CIH), Therapeutic Interactive Voice Response (TIVR), treatment as usual (TAU), mindfulness-oriented recovery enhancement (MORE), Pain, Enjoyment, and General Activity (PEG), * veteran or active duty participants.

### 3.2. Nonpharmacological Interventions Included in Studies

The NPIs used in the 39 studies were classified into nine intervention types: (1) combination of two or more NPIs [3,10,14,44,48,54,55,56,57,59,60,63,64,65,66], (2) educational programs [2,5,7,45,46,47], (3) noninvasive devices or digital technology [1,40,49,52,62], (4) cognitive behavioral therapy [29,42,61,67], (5) mindfulness [38,41,43], (6) acupuncture [30,53], (7) yoga [50,51], (8) hypnosis [39], and (9) chiropractic [58]. No studies included a NPI testing the effectiveness of music therapy or animal therapy. Only one study used tai chi/qigong, guided imagery, and hydrotherapy as a combination NPI (using two or more NPI)—not as a stand-alone intervention [63]. The combination NPI studies [38%, 15/39] included the following NPIs: ten studies [67%, 10/15] used cognitive behavioral therapy [3,10,14,48,54,55,57,59,64,66], nine studies [60%, 9/15] used a mindfulness technique [14,44,48,56,59,60,63,65,66], eight studies [53%, 8/15] used physical or occupational therapy [14,54,55,57,59,60,64,65], three studies [20%, 3/15] used acceptance and commitment therapy [3,65,66] and biofeedback [59,60,63], two studies [13%, 2/15] used chiropractic therapy [14,63], massage [14,63], and yoga [14,63], and one study [1/15, 7%] used hydrotherapy [14], breath practice [14], acupuncture [63], hypnosis [63], tai chi/qigong [63], and guided imagery [63]. Table 1 and supplementary material Table S1: Characteristics of Included Studies outlines the NPI type and intervention details for the studies included in the review.

#### 3.2.1. Combination NPI

Fifteen studies [38%, 15/39] (one RCT, one pilot RCT, twelve observational studies, and one secondary analysis) included a combination NPI treatment program approach for CNCP patients [3,10,14,44,48,54,55,56,57,59,60,63,64,65,66]. Each study that used a combination approach had a unique method of grouping one or more NPI. For example, one RCT combined mindfulness, evidence based integrative health approaches, and medical group visits [44]. One pilot RCT provided group-delivered CBT, mindfulness meditation, and mindfulness-based CBT [48]. Five studies [27%, 4/15] used an opioid medication taper protocol or guideline in addition to a combination NPI treatment program [54,55,59,64]. Six studies [40%, 6/15] demonstrated significant reduction in pain intensity and opioid use, concurrently [54,55,56,59,60,64]. Of these six studies, the following combination approaches were used: (1) combined CBT with occupational therapy and taper protocol [54], (2) combined CBT, group psychology, taper protocol, occupation therapy, and education [55], (3) combined CBT, psychotherapy, taper protocol [56], (4) combined occupational therapy, biofeedback, mindfulness and relaxation training, and education [60]; further, two studies [33%, 2/6] were investigated by the Mayo Clinic Comprehensive Pain Rehabilitation Center, an outpatient interdisciplinary pain rehabilitation program that incorporates physical/occupational therapy, biofeedback and relaxation techniques, stress management, and education [59,64]. Table 2 below details the specific components used in each combination NPI study, as well as identifies the six studies that successfully reduced both pain outcomes and opioid use. Additionally, Table 2 spotlights the combination NPI studies that synergistically used an integrated approach [73%, 11/15] compared to the “bag of tricks” tactic [27%, 4/15] that offers several interventions without a cohesive rational or clear mechanism of action.

#### 3.2.2. Educational Programs

Six studies [15%, 6/39] (four RCTs, one pilot RCT, and one observational study) included an educational program approach for adults with CNCP and opioid use [2,5,7,45,46,47]. These educational programs included self-management skills, opioid medication management and tapering guidance, guided imagery, patient–provider communication, theory-based programs, pain neuroscience, cognitive tools, nonopioid alternatives, biopsychosocial model, and pain management techniques. Sandhu et al. (2023) included a taper protocol in addition to the educational intervention [2]. Three studies [50%, 3/6] demonstrated a significant reduction in pain intensity and opioid use [45,46,47].

#### 3.2.3. Noninvasive Devices or Digital Technology

Five studies [13%, 5/39] (two RCTs, one pilot RCT, and one observational study) included a noninvasive device or digital technology for adults with CNCP and opioid use [1,40,49,52,62]. Garcia et al. (2021) investigated the effectiveness of a virtual reality (VR) program on adults with chronic low back pain [40]. Naylor et al. (2010) assessed a digital technology-based intervention, Therapeutic Interactive Voice Response (TIVR), an automated tool that participants use to interact with a computer through the medium of a telephone using the touch-tone keypad. TIVR was developed for the maintenance and enhancement of CBT skills [52]. Nelli et al. (2023) tested three visual light spectrum-based intervention arms: clear eyeglasses (control), green eyeglasses, or blue eyeglasses for adults with fibromyalgia [49]. Trinh et al. (2023) examined the effects of the H-Wave device stimulation (HWDS), a noninvasive, transcutaneous electrotherapy NPI that uses a proprietary “H waveform” associated with electromyography and the Hoffmann reflex to stimulate muscle fiber contraction for adults with CNCP [1]. Buchfuhrer et al. (2023) examined a Tonic Moto Activation (TOMAC) noninvasive device stimulation—two therapy units are specifically placed, bilaterally, over the peroneal nerve at the fibula bone for refractory restless legs syndrome chronic pain [62]. TOMAC produces a current controlled, charged balance 40,000 Hz stimulation waveform intensity > 40 milliamps. Two devices, the TIVR and H-Wave, demonstrated reductions in pain intensity and opioid use, simultaneously [1,52]. One digital technology, TIVR, demonstrated a significant improvement in pain outcomes and opioid cessation compared to usual care [1].

#### 3.2.4. Cognitive Behavioral Therapy (CBT)

Four studies [10%, 4/39] (two RCTs, one observational study, and one secondary analysis of a clinical trial) included a cognitive behavioral therapy intervention for adults with CNCP and opioid use [29,42,61,67]. Debar et al. (2022) assessed a CBT intervention that teaches pain self-management skills in 12 weekly 90 min groups delivered by a team (behaviorist, nurse, physical therapist, and pharmacist) versus usual care. Wartko et al. (2023) tested a CBT-based training intervention (STRIPE: Strategies to Improve Pain and Enjoy Life) compared to usual care for adults on long-term opioid therapy for CNCP [29]. Nilsen et al. (2010) examined a brief CBT intervention for adults with CNCP [61]. McCrae et al. (2020) examined the effect of CBT on sleep and opioid medication use in adults with fibromyalgia and insomnia [67]. No studies demonstrated a significant reduction in pain intensity and opioid use, concurrently. Nilsen et al. (2010) demonstrated a reduction in problematic codeine use after six sessions of CBT and opioid weaning, but no changes in reported pain intensity [61].

#### 3.2.5. Mindfulness

Three RCTs [8%, 3/39] assessed the effectiveness of Mindfulness Oriented Recovery Enhancement (MORE) for adults using opioids to treat CNCP [38,41,43]. Hudak et al. (2021) investigated the effects of 8 weeks of MORE (e.g., training in mindfulness, reappraisal, and savoring skills) for coping with opioid cravings, pain, and negative affect. Participants in MORE demonstrated greater opioid use reduction over time than the control group [41]. Garland et al. (2022) used MORE (e.g., training in mindfulness, reappraisal, and savoring positive experiences) compared to a supportive psychotherapy (control group) across 8 weekly/2 h group sessions. Approximately 45% of the participants in the MORE group stopped misusing opioids and showed greater improvements in pain outcomes compared to the control group [43]. Garland et al. (2024) tested MORE in past and present military personnel with long-term opioid use to treat CNCP compared to supportive psychotherapy. MORE demonstrated efficacy in reducing opioid use by 20.7%, compared to a 3.9% opioid reduction with supportive psychotherapy [38]. Overall, MORE significantly reduced pain intensity, opioid use, and opioid cravings, simultaneously.

#### 3.2.6. Acupuncture

Two studies [5%, 2/39] (one RCT and one observational study) assessed the effectiveness of acupuncture on adults with CNCP and taking opioids [30,53]. Zheng et al. (2019) evaluated the effect of electroacupuncture for adults with CNCP who were taking prescribed opioids [30]. Davis et al.’s study (2018) was conducted to assess the impact of acupuncture treatment with up to 12 total treatments within a 60-day period [53]. Davis et al. (2018) demonstrated a significant improvement in pain intensity with 32% of patients using opioid medication reporting reductions in use following the intervention [53].

#### 3.2.7. Yoga

Two studies [5%, 2/39] (two RCTs) included a yoga intervention for adults with CNCP and opioid use [50,51]. Groessl et al. (2017) included a 12-week yoga intervention consisting of two 60 min instructor-led yoga sessions per week. The intervention was hatha yoga, consisting in physical yoga postures, movement sequences, and regulated breathing. Participants were randomized to either yoga or delayed yoga treatment [51]. Roseen et al. (2022) assessed a yoga intervention consisting of 12 weekly 75 min yoga classes versus an educational (control) group [50]. Each class included a yoga breathing exercise (Pranayama), discussion of yoga philosophical principles, yoga postures (Asanas), and deep relaxation (Svasana). Each participant received a copy of *The Back Pain Helpbook*. Groessl et al. (2017) demonstrated significant decreases in pain intensity compared to the delayed control group, and a reduction in opioid pain medications from 20% to 11% at the 12-week follow-up and to 8% at the 6-month follow-up [51].

#### 3.2.8. Hypnosis

Jensen et al. (2020) [3%, 1/39] (RCT) assessed the effectiveness of hypnosis interventions on adults with CNCP [39]. Participants were randomized to one of four conditions (60 min sessions of educational control, hypnosis intervention, traditional cognitive therapy, or hypnotic cognitive therapy). Researchers found no statistically significant differences in pain intensity or opioid use among the groups [39].

#### 3.2.9. Chiropractic

Passmore et al.’s study (2022) [3%, 1/39] (observational study), assessing the effectiveness of chiropractic treatment for chronic pain and opioid use, demonstrated significant decreases in reported pain intensity and opioid use after a course of chiropractic care [58]. Significantly fewer people who used opioids at baseline (*n* = 58) no longer did at discharge at 12 weeks (*n* = 15). This represented a 59% reduction in the number of patients using opioids after progressing through a course of chiropractic care [58].

### 3.3. Reported Effect Sizes

Of the 19 RCTs (including pilot RCTs), 79% [15/19] reported effect sizes for pain outcomes and/or opioid use. Of the 20 non-RCTs (including secondary analyses), 40% [8/20] reported effect sizes for pain outcomes and/or opioid use. For RCTs, the pain outcome effect size range is 1.1 [−0.5–0.60] and the opioid use outcome effect size range is 2.93 [−0.20–2.73]. For non-RCTs, the pain outcome effect size range is 1.34 [0.06–1.4] and the opioid use outcome effect size range is 2.49 [0.41–2.9].

### 3.4. Measures

The majority of the studies [89.7%, 35/39] measured pain intensity by using one or a combination of the following measures: Numerical Rating Scale, Brief Pain Inventory, Visual Analogue Scale, pain intensity and interference with enjoyment of life, general activity (PEG), Defense and Veterans Pain Rating Scale (DVPRS), Patient Reported Outcomes Measurement Information System (PROMIS), Multidimensional Pain Inventory (MPI), West Haven Yale Multidisciplinary Pain Inventory, Clinical Global Impression of Improvement, short form of the McGill Pain Questionnaire, and the Pain Symptoms subscale from the Treatment Outcomes in Pain Survey. To measure opioid use, researchers used one or a combination of the following measures: morphine milligram equivalent (MME), self-reported, medical chart review, pill counts/medication bottles, and urine toxicology. Other reported outcomes included opioid misuse and risk, impression of change, fatigue, physical function, sleep disturbance, emotional distress, post-traumatic stress symptoms, pain coping, pain self-efficacy, pain knowledge, disability, health status, anxiety, depression, and quality of life. The measures are valid and reliable (Table 1 and Table 3).

### 3.5. Assessment of Methodological Quality

The Mixed Methods Appraisal Tool (MMAT) [36] was used to assess the quality of the included studies based on each study’s reporting, external validity, internal validity, and power. All authors contributed to the discussion regarding the overall quality and risk of bias in the included studies. The MMAT score is out of a total of 29 points, and the average of all included articles was 23.8. The risk for bias was relatively low for the 19 included RCTs (Table 4). It must be noted that given the nature of the NPIs covered in this review, several of the included studies were not able to blind subjects to the interventions, which may have biased participants’ responses on self-reported measures. Some of the included studies lacked randomization and statistical power to detect significant effectiveness. Few studies had samples of participants with a low morphine milligram equivalent (MME), which may indicate some selection bias. Additionally, several studies did not have a sample of participants that was representative of the entire population from which they were recruited. Table 4 below outlines the details for the MMAT (see supplementary material Table S2: Methodological Quality Assessment for additional details).

## 4. Discussion

In this review, we aimed to identify and describe the scope of the literature on the impact of NPIs on opioid use and pain-related outcomes among adults with CNCP. This review shows there is a small to moderate body of literature demonstrating positive evidence that NPIs may help reduce the use of opioids and pain intensity concurrently [1,38,43,45,46,47,51,52,53,54,55,56,58,59,60,64], decrease opioid cravings [38,43], and improve function [1,43,47,51,52,53,54,55], self-efficacy and/or pain coping [46,52,56,60], pain catastrophizing [38,56,59,64], mood and/or stress [1,38,43,45,52,53,55,56,60,64], sleep [53], pain interference [53,56], health status [60], social isolation [53], and quality of life [47]. To our knowledge, the present scoping review is the first to evaluate the impact of NPIs for adults with CNCP and opioid use, specifically excluding studies with cannabis or cannabinoids, pharmacological, and/or invasive interventions. Overall, there is a large body of literature on the use of NPIs for CNCP treatment. However, when we narrowed the literature search to include studies that measured opioid use and excluded interventions focused on cannabinoids, pharmaceuticals, and pain related to cancer, palliative care, end-of-life care, sickle cell interventions, HIV, acute care, labor, and postoperative treatments, only 39 articles were found.

Nineteen studies [49%, 19/39] focused on individuals with chronic low back pain (cLBP), making cLBP the most common location of pain reported, which aligns with the extant literature showing cLBP is the most common reported and disabling conditions [4,40,48,57]. Similarly, another scoping review on the use of NPIs for U.S. Medicare population characteristics showed that 55% of the studies included in their review reported back pain [4]. Additionally, similar to the scoping review mentioned above [4], our scoping review only included two studies on acupuncture [30,53]; however, unlike our scoping review, this article did not examine and report the impact of NPIs on opioid use and pain intensity. Demographically, twenty-eight [72%, 28/39] studies reported race and ethnicity characteristics, but only four studies [10%, 4/39] included a sample of >50% underrepresented populations [44,51,65,66], which aligns with a similar review [4]. Three studies [8%, 3/39] tested NPIs for CNCP patients with problematic opioid use or opioid misuse [14,43,61]. The studies reported a duration of NPI treatment from 10 days to 22 months, with the most common duration being eight weeks—slightly similar to Hassan et al. (2019), who reported durations of 3 weeks to 21 months. According to the MMAT for quality assessment, the average score of all included articles was 23.8 out of a total of 29—this indicated similar concerns of study quality (e.g., internal and external validity) with another review [4], which reported a low risk for bias among RCTs, and poor methodology and external generalizability among non-RCTs. However, unlike the article cited above, which included 52% of studies that did not report actual opioid intake [4], our review attempted to avoid these confounding variables by excluding articles that did not exclusively measure or report opioid use.

This scoping review found nine NPI types: (1) combination of two or more NPIs, (2) educational programs, (3) noninvasive devices or digital technology, (4) cognitive behavioral therapy, (5) mindfulness, (6) acupuncture, (7) yoga, (8) hypnosis, and (9) chiropractic treatment. Similar to another review, combination NPIs (including multidisciplinary treatment programs using two or more NPIs) were most commonly studied and demonstrated significant clinical improvements in pain and opioid use [34]—particularly, our review found six studies demonstrating a significant reduction in pain intensity and opioid use, concurrently [54,55,56,59,60,64]. Additionally, our review found similar NPI types as the review mentioned above [34]—educational programs, digital technology (Therapeutic Interactive Voice Response), cognitive behavioral therapy, mindfulness, and acupuncture; however, our review also included studies on yoga [50,51], hypnosis [39], and chiropractic treatment [58]. Similar to the review by Hassan et al. (2019), Therapeutic Interactive Voice Response demonstrated a reduction in pain intensity and opioid use [52]; however, our review also found that the H-Wave device significantly reduced pain and opioid use [1]. Our findings are consistent with a recent review [68]; the emergence of noninvasive devices and digital health technologies for CNCP are promising interventions to reduce opioid-based analgesia.

Furthermore, our review found similar results to the review by Hassan et al. (2019) regarding CBT and mindfulness. Our review found four studies that included a CBT intervention [29,42,61,67]—no significant decrease in pain and opioid use, concurrently, was observed. However, one study in our review demonstrated a reduction in problematic codeine use after six sessions of CBT and opioid weaning, but no changes were observed in reported pain intensity [61]. For mindfulness, Hassan et al. (2019) found that one study on Mindfulness Oriented Recovery Enhancement (MORE) reduced pain and the desire for opioids. However, our review included three studies that assessed the effectiveness of MORE for adults with CNCP [38,41,43]. Strong evidence supports the effectiveness of MORE in reducing pain intensity, opioid use, and opioid cravings, concurrently. Our review only found two studies [5%, 2/39] that assessed the effectiveness of acupuncture on adults with CNCP and taking opioids [30,53]— one study demonstrated a significant improvement in pain intensity and a reduction in opioid medication use following the intervention [53]—similar findings were also reported in other reviews [34,69]. Resembling our review, the use of education programs, especially for medication weaning or opioid tapering, were commonly used in other reviews [34,69]—our review found three studies that significantly reduced pain intensity and opioid use [45,46,47]. Only 44% of the studies in this review clearly reported details regarding adverse events. This is consistent with prior reviews, and there is a lack of evidence about the possible harms related to study participation [34,69].

In addition to references [34,69], the reprint review by Cai et al. (2024) includes three similar studies that our review contains [2,29,41]; however, the authors inclusion criteria (e.g., pharmaceuticals), setting (e.g., explicitly aimed to investigate the primary care setting), and selected interventions (e.g., cannabis) differ greatly compared to the scope and aim of our scoping review that explicitly addresses NPIs. Furthermore, compared to Cai et al. (2024), our review findings provide updated (2024 studies) and more robust literature (18 RCTs), and greater detail regarding secondary pain-related outcomes and participant characteristics (e.g., CNCP diagnosis, race, ethnicity). Similar to our review, Cai et al. (2024) emphasizes the careful consideration needed related to using cannabis long-term as an alternative for opioid therapy to treat CNCP, including the potential risks of a reduced educational level, mental health concerns, and residual cognitive complications [70].

It is worth noting that no studies in this review included an NPI testing the effectiveness of music therapy or animal therapy to reduce pain and opioid use. Only one study used tai chi/qigong, guided imagery, and hydrotherapy as a combination NPI (using two or more NPIs simultaneously)—none were tested as a stand-alone intervention [63]. Strong evidence supports the effectiveness of tai chi/qigong in reducing anxiety [71,72,73], pain [74,75,76], and improving mood [77], sleep, self-efficacy [78], and physical function [79] in individuals with CP. However, as revealed in other reviews, RCTs investigating the effectiveness of tai chi/qigong to manage pain and reduce opioid use are lacking [4,72]. There is a critical need for rigorous studies of NPIs to reduce opioid use and more effectively treat pain conditions while limiting the risk of opioid adverse events for CNCP individuals.

### Limitations

The present scoping review followed recommendations for rigorous reviews with four independent reviewers conducting the literature search, extracting data, and assessing study quality. To identify as many applicable studies as possible and reduce the risk of bias for this review, a thorough and highly sensitive search strategy was employed. There were several limitations to this review study. This review aimed to review and synthesize all the available evidence on the selected criteria, irrespective of study design. Our analyses were limited to full-text articles written in English, which may have omitted relevant high-quality studies and resulted in a bias against articles published in other languages. Potentially relevant articles may have been missed in the search if they did not specifically mention our selected search terms in their abstract/subject heading/title.

For the observational studies and secondary analyses, the lack of comparison groups and the quasi-experimental or single-arm nature of the studies may have affected their external generalizability. The high attrition rate at follow-up was an additional limitation, which may have introduced reporting bias and reduced the validity of the results. Additionally, there was a limitation to generalizability due to small sample sizes, homogeneous samples of White males, no control group or placebo, and it is unclear which technique or parts of the intervention were responsible for the reported results from several inter/multidisciplinary studies. Measures of pain and opioid use were self-reported using validated measures, and responses may be influenced by unmeasured factors. These confounding variables are important factors in assessing the quality of evidence.

There is a common thread among these studies; they did not employ the most powerful analytical procedures and used repeated measures in longitudinal studies, though multilevel modeling is widely accepted as a better method than repeated measures [80,81]. Additionally, many studies under review used combination NPI approaches. Without controlling and partitioning the effects of the treatments, we cannot indicate which particular component (e.g., yoga, meditation, CBT, etc.) is more helpful. A thorough collection of information and data for all interventions (including dose, duration, adherence, attrition, and adverse events) is imperative for replicating and drawing widespread recommendations about intervention effects [34]. NPIs include a wide range of psychological (e.g., cognitive restructuring, problem-solving skills-training, meditation), physical (e.g., progressive muscle relaxation, acupuncture), or blended psychological and physical (e.g., tai chi, yoga) approaches to manage or reduce stress, improve coping, and promote self-management [82]. Hence, it is important to select outcome measures intentionally and cautiously. The selected outcomes for CNCP management must reflect the core philosophy of whole-person health [34]. This scoping review found a gap in the literature on the cost effectiveness and efficacy of NPIs, especially for low income, racially, and ethnically diverse patients. Access to NPIs is challenging due to disparities in access, as these approaches are located far from rural areas, often require out of pocket payment, lack insurance reimbursement, and are rarely offered as treatment options to underrepresented populations [44].

Recommendations for future research include conducting more RCTs to investigate NPIs to treat CNCP for individuals with and without opioid use disorder. The use of guided imagery, tai chi/qigong, animal-assisted therapy, and music therapy are used in practice but have not been studied systematically. Additionally, conducting dismantling studies of effective combination NPI treatments, in order to identify their most influential effect or “active ingredients”, are warranted. Furthermore, future research should consider the retention in care as an additional outcome beyond pain and opioid use—this remains a serious concern in managing medications for opioid use disorder. Given the severity of the opioid crisis, it is critical to investigate whether some of the NPIs could be pragmatically combined or integrated with medications for opioid use disorder to improve treatment adherence and reduce relapse potential—CBT is most commonly used, but other NPIs might be more beneficial.

## 5. Conclusions

This review suggests that mindfulness (e.g., Mindfulness Oriented Recovery Enhancement), yoga, certain educational programs, devices or digital technology (e.g., H-Wave, Therapeutic Interactive Voice Response), chiropractic therapy, and combination NPIs may be effective for managing pain-related symptoms and reduce opioid use among adults with CNCP treated with long-term opioids. Across the studies reviewed, these specific NPIs were associated with improvements in quality of life, function, self-efficacy and coping, mood, and stress. However, other NPI approaches were not effective (e.g., hypnosis, virtual reality device, and cognitive behavioral therapy as a standalone intervention). It is important that clinicians and patients are informed of alternate evidence-based interventions to manage pain-related symptoms and safely reduce opioid use. The results of this review must be interpreted with caution as there were limited studies that investigated NPIs with rigorous study designs, diverse samples, and adequate descriptions of intervention features. More research is needed to further elucidate the effect of NPIs to treat pain and mitigate opioid use among adults with CNCP, particularly those from underrepresented groups.

## Figures and Tables

**Table 2:** Combination Nonpharmacological Interventions.

NPI	Gardiner, 2019	Day, 2019	Moffat, 2023	Zeliadt, 2022	Huffman, 2019	Townsend, 2008	Ward, 2022	Van Der Merwe, 2021	Hooten, 2007	Schumann, 2020	Gibson, 2020	Van Hoof, 2012	Gilliam, 2020	Barrett, 2021	Miller- Matero, 2022
Mindfulness	X	X					X							X	X
Relaxation Techniques						X			X		X				
CBT		X			X	X		X		X	X	X	X	X	X
Education	X		X	X	X	X	X	X	X					X	
Biofeedback				X		X			X						
Yoga				X											
Audit and Feedback			X												
Taper Protocol			X		X			X		X			X		
Physical or Occupational Therapy or Movement					X	X	X		X	X	X	X	X		
Guided Imagery				X											
Group Visits	X	X			X									X	
Hypnosis				X											
Acupuncture				X											
ACT							X							X	X
Psychotherapy					X										
Stress Management						X			X						
Chiropractic				X							X				
Tai Chi/Qigong				X											
Meditation		X		X							X				
Massage				X			X				X				
Whole Health Coaching				X											
Hydrotherapy											X				
Breathing Practices											X				
Device or Digital Technology	X														
Reduced Pain and Opioid Use?	N	N	N	N	Y	Y	N	Y	Y	Y	N	N	Y	N	N
Integrated Approach?	Y	Y	N	N	Y	Y	Y	Y	Y	Y	N	Y	Y	Y	N

Abbreviations: nonpharmacological intervention (NPI), cognitive behavioral therapy (CBT), acceptance and commitment therapy (ACT), Y = yes, N = no, “X” = study included NPI.

**Table 3:** Additional Outcomes Assessed in Studies.

First Author, Year	Additional Measures	Additional Results
Garcia, 2021	Pain Interference with Activity, Sleep, Mood, and Stress (DVPRS-II, PROMIS), Pain Catastrophizing Scale (PCS), Pain Efficacy (PSEQ-2), Chronic Pain Acceptance (CPAQ-8), Patient’s Global Impression of Change, Satisfaction with VR Device Use, Cybersickness, Over-the-Counter Analgesic Medication Use	EaseVRx intervention decreased pain-related interference with activity, mood, and stress, and nonopioid medication use. Pain catastrophizing, pain self-efficacy, and pain acceptance did not reach statistical significance for either group.
Jensen, 2020	Pain Interference (BPI), Depressive (PHQ-8), Global Impression of Change (IMMPACT), Satisfaction (PGATS)	All 4 treatment groups showed improvements on pain-related interference and depressive symptoms, with some return to pre-treatment levels at 12-month follow-up.
Zheng, 2019	Medication Quantification Scale III was used to quantify nonopioid medications, Unpleasantness was measured with a 0–20 Numerical Rating Scale, Depression (BDI), Quality of Life (SF-36), Disability (RMDQ), Perception of Electroacupuncture Treatment Questionnaire	There were no significant differences found across the treatment groups on mental health, feelings of unpleasantness, nonopioid medication doses, disability, and opioid-related adverse events.
Garland, 2022	Pain Interference (BPI), Emotional distress (DASS), Opioid Misuse and Cravings (DMI, COMM)	MORE group experienced greater reductions in pain-related functional interference and lower emotional distress and opioid cravings than the supportive psychotherapy group.
Hudak, 2021 *	Self-referential Processing (NADA-state, PBBS)	MORE group demonstrated significantly increased alpha and theta power and increased frontal midline theta coherence compared to the control group—neural changes with altered self-referential processing were noted.
Wilson, 2023	Opioid Misuse (COMM), Global Health (PROMIS), Pain Knowledge (The Pain Knowledge Questionnaire), Pain Self-Efficacy (PSEQ), Pain Coping (CSQ-R)	No significant effect found from baseline to 10-month posttest for COMM and Global Health. Improvements were found in pain knowledge, pain self-efficacy, and pain coping.
Garland, 2024 *	Emotional Stress (DASS), Post-Traumatic Stress Disorder Checklist—Military Version, Pain Catastrophizing subscale of the Coping Strategies Questionnaire, the Snaith–Hamilton Anhedonia and Pleasure Scale, the positive affect subscale of the Positive and Negative Affect Schedule, the Cognitive Reappraisal of Pain Scale, and Nonreactivity Subscale of the Five Facet Mindfulness Questionnaire, Opioid Cravings (COMM)	MORE group reduced opioid use while maintaining pain control and preventing mood disturbances. MORE group reduced opioid cravings, opioid cue reactivity, anhedonia, pain catastrophizing, and opioid attentional bias and increased positive affect more than the control group.
DeBar, 2022	Roland–Morris Disability Questionnaire (RMDQ)	CBT intervention sustained larger reductions in pain related disability.
Gardiner, 2019	Depression (PHQ-9), Patient Activation Measure, Health-related Quality of Life (short form 12 Health Survey version 2: SF-12), Opioid Misuse (COMM)	Significant differences between the intervention and control group for activation and opioid misuse. No differences in depression at any time point. At 21 weeks, the intervention group had higher quality of life compared with the control group
Wartko, 2023	Pain Self-Efficacy (PSEQ), Depression (PHQ-8), Generalized Anxiety (GAD-7), Patient Global Impression of Change, Prescription Opioid Difficulties Scale, Prescription Opioid Misuse Index	No significant differences between intervention and usual care were found for any of the secondary outcomes.
Groessl, 2017 *	Roland–Morris Disability Questionnaire (RMDQ)	Improvements in disability scores did not differ between the two groups at 12 weeks, but yoga showed greater reductions in disability scores than delayed treatment group at 6 months.
Roseen, 2022 *	Post-Traumatic Stress Symptoms (PCL-C), Roland–Morris Disability Questionnaire (RMDQ)	No significant differences between intervention and education were found for secondary outcomes.
Sandhu, 2023	Patient-Reported Outcomes Measurement Information System (PROMIS-PI-SF-8a), Short Opioid Withdrawal Scale (SHOWS), Health-related Quality of Life (SF-12v2 health survey and EuroQol 5-dimension 5-level), Sleep Quality (Pittsburgh Sleep Quality Index), Emotional Wellbeing (HADS), Pain Self-Efficacy (PSEQ)	At 4-month follow-up, the education intervention showed significant improvements in mental health, pain self-efficacy, and health-related quality of life, but did not show improvements at any other data collection time point. No statistically significant between-group differences in opioid withdrawal symptoms, sleep quality, or pain interference were found.
Does, 2024	Depression (PHQ-9), Quality of Life, Health, and Functional Status (PROMIS), Patient Activation Measure (PAM-13)	The intervention demonstrated less moderate/severe depression symptoms and higher overall health and function status. The intervention had no effect on activation scores at 12 months.
Naylor, 2010	Function/Disability from the Treatment Outcomes in Pain Survey, Depression (BDI), Pain Coping (CSQ).	TIVR intervention group demonstrated improved coping, depression symptoms, function, and disability, compared to the standard follow-up group.
Nielssen, 2019	Depression (PHQ9), Anxiety (GAD-7)	Reduction in opioid consumption was strongly associated with decreases in anxiety and depression symptoms.
Day, 2019	Physical Function, Depression, and Pain Interference (PROMIS)	MBCT group improved significantly more than MM group on pain interference, physical function, and depression symptoms. MBCT and CT group did not differ significantly on any of the measures.
Spangeus, 2023	Health-related Quality of Life (EQ-5D-3L, RAND-36, Qualeffo-41), Static and Dynamic Balance Tests, Fall Risk and Physical Activity (FES-I), Theoretical Knowledge (open-ended questions)	Significant improvements were found for quality of life, balance, tandem walking backwards, and theoretical knowledge. These changes were maintained at the 1-year follow-up.
Nelli, 2023	NA	NA
Moffat, 2023 *	NA	NA
Zeliadt, 2022 *	NA	NA
Huffman, 2019	Pain-related Functional Impairment (PDI), Depression and Anxiety (DASS)	Intervention showed significant pre-post treatment improvements in functional impairment, depression, and anxiety symptoms.
Townsend, 2008	Health Status (SF-36), Pain Catastrophizing Scale (PCS), Depression (CES-D)	Significant improvements were found on health status, pain catastrophizing, and depression symptoms following treatment and six-month post-treatment irrespective of opioid status at admission.
Ward, 2022 *	Depression (PHQ9), VA Stratification Tool for Opioid Risk Mitigation (STORM)	Reduced depression scores in the post-treatment year were found in the engaged group. EVP showed a 65% lower mortality risk compared to the untreated group.
Van Der Merwe, 2021 *	Pain Interference (BPI), Pain Catastrophizing Scale (PCS), Mood (CORE), Post-traumatic Stress Symptoms (Impact of Events Scale: IES-6), Self-Efficacy and Confidence (PSEQ)	Pain management program significantly improved pain-related interference, mood, self-efficacy, and confidence, post-traumatic stress symptoms, and pain catastrophizing.
Hooten, 2007	Health Status (SF-36), Pain Coping (CSQ), Depression (CES-D)	Health status, coping, and depression scores demonstrated improvement with the intervention.
Davis, 2018	Pain Interference, Fatigue, Physical Function, Sleep Disturbance, Emotional Distress—Anxiety, Emotional Distress—Depression, and Social Isolation Short Forms (PROMIS)	Significant improvements were found in pain-related interference, physical function, fatigue, anxiety, depression, sleep disturbance, and social isolation.
Schumann, 2020	Pain Catastrophizing Scale (PCS), Depressive symptoms (CES-D, PHQ-9), Quality of Life (Medical Outcomes Study 36-Item Short Form Survey)	Significant treatment effects with large effect sizes were observed for all outcome measures at post-treatment and 6-month follow-up.
Gibson, 2020 *	Pain Catastrophizing Scale (PCS), Current Opioid Misuse Measure (COMM), Patient Treatment Satisfaction Scale (PTSS)	Significant decrease in pain-related interference, pain catastrophizing, pain magnification, pain helplessness, and opioid misuse were found.
Van Hoof, 2012	Roland and Morris Disability Questionnaire (RMDQ), SF36 PCS Short Form 36 Physical Component Scale, SF36 MCS Short Form 36 Mental Component Scale, pain disturbance of ADLs (0–100 scale)	For the 1 and 2-year follow-up, only pain disturbance of ADLs significantly improved: *df* (1,84), t = 2.57, *p* = 0.01.
Gilliam, 2020	PTSD Checklist with a brief Criterion A assessment (PCL-5), Pain Catastrophizing Scale (PCS), Depression (PHQ-9), Physical performance measures	Intervention showed significant improvements in PTSD, depression, physical performance, and pain outcomes.
Trinh, 2023	Depression (PHQ9), Anxiety (GAD-7), Pain Disability Questionnaire	Intervention showed a 24.4% reduction in depression, 31% reduction in anxiety, and significant improvement in function/disability.
Passmore, 2022	NA	NA
Buchfuhrer, 2023	NA	NA
Barrett, 2021	Pain Interference (BPI), Pain willingness and activity engagement (CPAQ), Depression (PHQ-9)	No significant changes in pain interference, but significant improvements in pain willingness, activity engagement, and depression were found.
Matyac, 2022	Opioid Risk (ORT), Pain Catastrophizing (PCS)	The program showed reduction in pain catastrophizing and pain scores. Combining data from opioid risk and data on sleep apnea, the results showed that 31% of participants were at high risk of opioid overdose.
Nilsen, 2010	Health-related Quality of Life (SF-36), Neurocognitive Tests	Neuropsychological functioning improved on some tests; others remained unchanged. Opioid use decreased without significant reduction in quality of life.
McCrae, 2020	NA	NA
Miller-Matero, 2022	Pain Interference (BPI), Pain Catastrophizing (PCS), Depressive Symptoms (HADS)	Intervention showed decreases in pain catastrophizing and depression symptoms. There were significant improvements in pain-related interferences.

Abbreviations: cognitive behavioral therapy (CBT), cognitive therapy (CT), mindfulness meditation (MM), mindfulness-based cognitive therapy (MBCT), electronic medical record (EHR), Veterans Health Administration (VA), Biopsychosocial Integrated Pain Team (IPT), Brief Pain Inventory (BPI), Pain Catastrophizing Scale (PCS), Current Opioid Misuse Measure (COMM), Pain Treatment Satisfaction Scale (PTSS), Patient Health Questionnaire (PHQ-8 and PHQ-9), Generalized Anxiety Disorder Scale (GAD-7), Initiative on Methods, Measurement, and Pain Assessment in Clinical Trials (IMMPACT), participant global satisfaction with treatment (PGATS), Patient-Reported Outcomes Measurement Information System (PROMIS), Center for Epidemiologic Studies Depression Scale (CES-D), Defense and Veterans Pain Rating Scale (DVPRS-II), Chronic Pain Acceptance Questionnaire (CPAQ-8), Roland–Morris Disability Questionnaire (RMDQ), System Usability Scale (SUS), Coping Strategies Questionnaire-Catastrophizing subscale (CSQ-C), Short Form-36 Health Status Questionnaire (SF-36), Nondual Awareness Dimensional Assessment (NADA-state), Perceived Body Boundaries Scale (PBBS), Pain Disability Index (PDI), Pain Self-Efficacy Scale (PSEQ), Patient Activation Measure (PAM-13), Short Opioid Withdrawal Scale (SHOWS), tonic motor activation (TOMAC), acceptance and commitment therapy (ACT), Clinician Global Impression of Improvement (CGI-I), Empower Veterans Program (EVP), complementary and integrative health therapies (CIH), Therapeutic Interactive Voice Response (TIVR), treatment as usual (TAU), mindfulness-oriented recovery enhancement (MORE), opioid risk (ORT), Stratification Tool for Opioid Risk Mitigation (STORM), Impact of Events Scale (IES-6), Beck Depression Inventory (BDI), Drug Misuse Index (DMI), Post Traumatic Stress Symptoms (PCL-C), Post Traumatic Stress Disorder (PTSD), Fall Risk and Physical Activity (FES-I), European Quality of Life (EQ-5D-3L and RAND-36), Quality of Life Questionnaire in the European Foundation for Osteoporosis (Qualeffo-41), Hospital Anxiety and Depression Scale (HADS); * veteran or active duty participants.

**Table 4:** Methodological Quality Assessment.

First Arthur, Year	Total Quality Index Score (Range, 0–29 Points)
Garcia, 2021	29
Jensen, 2020	29
Zheng, 2019	29
Garland, 2022	27
Hudak, 2021	27
Wilson, 2023	27
Garland, 2024	27
DeBar, 2022	27
Gardiner, 2019	26
Wartko, 2023	26
Groessl, 2017	26
Roseen, 2022	26
Sandhu, 2023	25
Does, 2024	24
Naylor, 2010	24
Nielssen, 2019	22
Day, 2019	24
Spangeus, 2023	23
Nelli, 2023	24
Moffat, 2023	21
Zeliadt, 2022	23
Huffman, 2019	23
Townsend, 2008	23
Ward, 2022	23
Van Der Merwe, 2020	23
Hooten, 2007	22
Davis, 2018	23
Schumann, 2020	23
Gibson, 2020	22
Van Hooff, 2012	17
Gilliam, 2020	21
Trinh, 2023	23
Passmore, 2022	23
Buchfuhrer, 2023	21
Barrett, 2023	23
Matyac, 2022	20
Nilsen, 2010	19
McCrae, 2020	21
Miller-Matero, 2022	23

Table 4 above is condensed. For full details, see supplementary material Table S2: Methodological Quality Assessment for additional details.

## Data Availability

The original contributions presented in the study are included in the article/Supplementary Materials; further inquiries can be directed to the corresponding author.

## Supplementary Materials

The following supporting information can be downloaded at: https://www.mdpi.com/article/10.3390/ijerph21060794/s1, Table S1: Characteristics of Included Studies; Table S2: Methodological Quality Assessment.

## Appendix A

**Table A1:** Preferred Reporting Items for Systematic Reviews and Meta-Analyses extension for Scoping Reviews (PRISMA-ScR) Checklist.

Section	Item	Prisma-ScR Checklist Item	Reported on Page #
**Title**			
Title	1	Identify the report as a scoping review.	1
**Abstract**			
Structured summary	2	Provide a structured summary that includes (as applicable) background, objectives, eligibility criteria, sources of evidence, charting methods, results, and conclusions that relate to the review questions and objectives.	1
**Introduction**			
Rationale	3	Describe the rationale for the review in the context of what is already known. Explain why the review questions/objectives lend themselves to a scoping review approach.	1–3
Objectives	4	Provide an explicit statement of the questions and objectives being addressed with reference to their key elements (e.g., population or participants, concepts, and context) or other relevant key elements used to conceptualize the review questions and/or objectives.	3
**Methods**			
Protocol and registration	5	Indicate whether a review protocol exists; state if and where it can be accessed (e.g., a Web address); and if available, provide registration information, including the registration number.	2–3
Eligibility criteria	6	Specify characteristics of the sources of evidence used as eligibility criteria (e.g., years considered, language, and publication status), and provide a rationale.	3
Information sources	7	Describe all information sources in the search (e.g., databases with dates of coverage and contact with authors to identify additional sources), as well as the date the most recent search was executed.	4
Search	8	Present the full electronic search strategy for at least 1 database, including any limits used, such that it could be repeated.	28–29
Selection of sources of evidence†	9	State the process for selecting sources of evidence (i.e., screening and eligibility) included in the scoping review.	3
Data charting process‡	10	Describe the methods of charting data from the included sources of evidence (e.g., calibrated forms or forms that have been tested by the team before their use, and whether data charting was performed independently or in duplicate) and any processes for obtaining and confirming data from investigators.	4
Data items	11	List and define all variables for which data were sought and any assumptions and simplifications made.	3
Critical appraisal of individual sources of evidence§	12	If performed, provide a rationale for conducting a critical appraisal of included sources of evidence; describe the methods used and how this information was used in any data synthesis (if appropriate).	3
Synthesis of results	13	Describe the methods of handling and summarizing the data that were charted.	4
**Results**			
Selection of sources of evidence	14	Give numbers of sources of evidence screened, assessed for eligibility, and included in the review, with reasons for exclusions at each stage, ideally using a flow diagram.	5
Characteristics of sources of evidence	15	For each source of evidence, present characteristics for which data were charted and provide the citations.	5
Critical appraisal within sources of evidence	16	If performed, present data on critical appraisal of included sources of evidence (see item 12).	23–24
Results of individual sources of evidence	17	For each included source of evidence, present the relevant data that were charted that relate to the review questions and objectives.	6–11
Synthesis of results	18	Summarize and/or present the charting results as they relate to the review questions and objectives.	13–18
**Discussion**			
Summary of evidence	19	Summarize the main results (including an overview of concepts, themes, and types of evidence available), link to the review questions and objectives, and consider the relevance to key groups.	24–26
Limitations	20	Discuss the limitations of the scoping review process.	26–27
Conclusions	21	Provide a general interpretation of the results with respect to the review questions and objectives, as well as potential implications and/or next steps.	27
**Funding**			
Funding	22	Describe sources of funding for the included sources of evidence, as well as sources of funding for the scoping review. Describe the role of the funders of the scoping review.	27

## Appendix B

The following search strategy was developed in PubMed and adapted to each database: (((nonpharmacological*[Title/Abstract OR “non-pharmacological*”[Title/Abstract] OR “alternative and complementary”[Title/Abstract] OR “complementary and alternative”[Title/Abstract] OR “Complementary Therapies”[Mesh] OR “alternative medic*”[Title/Abstract] OR “complementary therap*”[Title/Abstract] OR “complementary medic*”[Title/Abstract]) OR (“Cognitive Behavioral Therapy”[Mesh] OR “cognitive behavioral”[Title/Abstract] OR CBT[Title/Abstract] OR biopsychosocial[Title/Abstract])) OR (chiropract*[Title/Abstract] OR osteopath*[Title/Abstract] OR neurostimula*[Title/Abstract] OR biofeedback[Title/Abstract] OR neurofeedback[Title/Abstract] OR “neuro feedback”[Title/Abstract] OR acupuncture[Title/Abstract] OR acupressure[Title/Abstract] OR “tai chi”[Title/Abstract] OR “tai ji”[Title/Abstract] OR massage[Title/Abstract] OR yoga[Title/Abstract] OR qigong[Title/Abstract] OR meditation[Title/Abstract] OR “guided imagery”[Title/Abstract] OR “music therapy”[Title/Abstract] OR “art therapy”[Title/Abstract] OR “therapeutic touch”[Title/Abstract])) AND (“Opioid-Related Disorders”[Mesh] OR “opioid addiction”[Title/Abstract] OR “opiate addiction”[Title/Abstract] OR “opiate abuse”[Title/Abstract] OR “opioid abuse”[Title/Abstract] OR “opiate dependen*”[Title/Abstract] OR “opioid dependen*”[Title/Abstract] OR “Analgesics, Opioid”[Mesh] OR “Chronic Pain”[Mesh] OR “chronic pain”[Title/Abstract])) AND(“Treatment Outcome”[Mesh] OR effect*[Title/Abstract] OR efficac*[Title/Abstract] OR result*[Title/Abstract] OR outcome*[Title/Abstract] OR improve*[Title/Abstract] OR control*[Title/Abstract] OR diminish*[Title/Abstract] OR respond[Title/Abstract] OR respons*[Title/Abstract])).
