# Supplementary material for: The Impact of Nonpharmacological Interventions on Opioid Use for Chronic Noncancer Pain: A Scoping Review
Source: Int J Environ Res Public Health. 2024 Jun 18;21(6):794. doi: 10.3390/ijerph21060794 (PMC11203961; doi:10.3390/ijerph21060794)
Supplement: Supplementary file 1 [file ijerph-21-00794-s001.zip › ijerph-3037316-supplementary.pdf]

Table S1. Characteristics of Included Studies in the Scoping Review

First Author and Year	Design	N	Mean Age	Female %	Race and Ethnicity	Pain Type	NPI Type and Intervention Details	Pain Intensity and Opioid Use Measures	Pain Intensity and Opioid Use Results	Limitations
Garcia (2021)	RCT	179	51.5	76.5%	White (90.5%)<br>Black (3.4%)<br>Multiracial (2.8%)<br>Asian (1.7%)<br>Other (1.1%)<br>Missing (0.6%)	Chronic low back pain (cLBP)	NPI Type: Device<br>Duration: 56 days<br>Details: Virtual reality (EaseVRx) combines biopsychosocial education, diaphragmatic breathing training, relaxation response exercises that activate the parasympathetic nervous system, and executive functioning games to provide a mind–body approach to treat CNCP. Sham VR (control) group received 2D nature footage.	Pain: Defense and Veterans Pain Rating Scale (DVPRS)<br><br>Opioid use: Self-reported and converted to morphine milligram equivalent (MME)	Pain: Pain intensity reduced by an average of 42.8% for the virtual reality (EaseVRx) group and 25% for the sham virtual reality group.<br><br>Opioid use: Did not reach statistical significance for either group.	Self-reported measures. For opioid use - unreported dose/frequency was marked as standard opioid dosage. Study sample was mostly White race, college educated, and internet savvy, which limits generalizability.
Jensen (2020)	RCT	173	NA<br><br>(age range 24-81)	58.9%	White (78.0%)<br>Black (8.1%)<br>Asian (4.0%)<br>Other (4.0%)<br>Multiracial (4.6%)<br>Hispanic/ Latino (2.3%)	cLBP, spinal cord injury, multiple sclerosis acquired amputation, muscular dystrophy	NPI Type: Hypnosis<br>Duration: 4 total sessions<br>Details: Participants were randomized and offered to receive 4 individual 60-minute sessions of an intervention. Education control intervention (control), hypnosis intervention, traditional cognitive therapy, and hypnotic cognitive therapy.	Pain: Numeric Rating Scale<br><br>Opioid use: Self-reported and converted to MME	Pain: No statistically significant between-group differences on omnibus test for pain intensity. On average, pain intensity reduced between pre- vs. post-treatment for all groups.<br><br>Opioid use: No changes in opioid use were found.	Self-reported measures. Control condition was associated with improvement in study outcomes. The number of treatment sessions (4) tested was low relative to the number of sessions often tested in clinical trials of psychosocial chronic pain interventions. It is possible that the efficacy of one or more of the treatments might have been found to be greater if more treatment sessions were offered.
Zheng (2019)	RCT	108	NA<br><br>(age range 18-85)	56.5%	NA	Chronic musculoskeletal pain	NPI Type: Acupuncture<br>Duration: 12 weeks<br>Details: All participants received pain and medication management education. Participants were asked to taper their opioid dosage by 30% in week 8, 50% by week 11, and 75% to 100% by week 14, as long as their pain did not get worse. Participants were randomly allocated to electroacupuncture, sham electroacupuncture, or education alone for 12 weeks. Twelve needles were used for each session, consisting in four formula points for electroacupuncture.	Pain: Visual Analogue Scale<br><br>Opioid use: Self-reported and converted to MME	Pain: No group differences were found in pain intensity. No changes in pain intensity were found over time.<br><br>Opioid use: Opioid use: Opioid use reduced by 20.5% (*p*<0.05) and 13.7% (*p*<0.01) in the two acupuncture groups and by 4.5% in the education group post-treatment, but without any group differences. For follow-up, the education group had a 47% decrease in opioid use after a course of electroacupuncture.	Self-reported measures. The planned 120 participants were not attained due to difficulties in recruitment. This study had a relatively high dropout rate of 17%.
Garland (2022)	RCT	250	51.8	63.6%	White (87.2%)<br>Hispanic/ Latino (6.8%)<br>Other (6.0)	Back, osteoarthritis, fibromyalgia, neuropathic, cervical, extremity, migraine or headache, irritable bowel syndrome, interstitial cystitis, pelvic pain, other or unspecified	NPI Type: Mindfulness<br>Duration: 8 weeks<br>Details: The mindfulness-oriented recovery enhancement (MORE) and supportive psychotherapy interventions were delivered in primary care clinics to groups of 6 to 12 participants across 8 weekly 2-hour sessions. The manualized MORE intervention provided sequenced training in mindfulness, reappraisal, and savoring skills. Control group was supportive group (SG) psychotherapy.	Pain: Brief Pain Inventory (BPI)<br><br>Opioid use: Urine toxicologic screening, Self-reported, and converted to MME	Pain: The MORE group showed greater reductions in pain severity (between-group effect: 0.49; 95% CI, 0.17-0.81; *p*= 0.003) than the SG group.<br><br>Opioid use: The MORE group reduced opioid use more than the control group (between-group effect: 0.15 log mg; 95% CI, 0.03-0.27 log mg; *p*= 0.009). At 9-month follow-up, 22 of 62 participants (35.5%) in the MORE group reduced opioid use by at least 50%, compared to 11 of 69 participants (15.9%) in the control group (*p*=0.009). At 9 months, 36 of 80 participants (45.0%)	The discontinuation rate, which was similar to that of other clinical trials of psychosocial treatment for individuals using opioids (mean discontinuation rate of 42%). The lack of stratified randomization by long-term opioid therapy duration or the use of medications for opioid use disorder.

									in the MORE group were no longer misusing opioids compared with 19 of 78 participants (24.4%) in the control group.	
Hudak (2021)	RCT	62<br>*	59.3	14.5%	White (82.3%)<br>Hispanic/ Latino (4.8%)<br>Native American or American Indian (4.8%)<br>Black (3.2%)<br>Other (3.2%)	Back, leg, joints, neck/shoulders, other	<u>NPI type:</u> Mindfulness<br><u>Duration:</u> 8 weeks<br><u>Details:</u> The MORE intervention was delivered for 8 weeks, with 2-hour long group sessions led by a psychologist. Patients were provided training in mindfulness, reappraisal, and savoring skills as techniques to cope with opioid craving, pain, and negative affect. Participants were asked to engage in daily 15 min mindfulness sessions at home guided by an audio recording. Control group was supportive group (SG) psychotherapy.	<u>Pain:</u> NA<br><br><u>Opioid use:</u> Self-reported and converted to MME	<u>Pain:</u> NA<br><br><u>Opioid use:</u> Participants in MORE showed greater reduction in opioid use over time than the control group.	Participants in the study were instructed to take opioids as prescribed on the day of the experiment to prevent withdrawal-related cognitive and neurophysiological disturbances; the acute pharmacological effects of opioids may have influenced neurophysiological responses. The study had a modest sample size. It is possible that decreases in opioid dosing might have driven some of the observed EEG changes, rather than the reverse. There was no measure of pain intensity.
Wilson (2023)	RCT	402	56.7	69.4%	White (74.9%)<br>Black (17.9%)<br>Other (7.2%)<br>Hispanic/ Latino (1.2%)	Arthritis, back, fibromyalgia, joint, migraines, neck, nerve pain/ neuropathy	<u>NPI Type:</u> Educational program<br><u>Duration:</u> 8 weeks<br><u>Details:</u> The E-Health online educational program intervention was delivered for 8 weeks, approximately 1-2 hours per week and includes these concepts: 1) an individual's active role in pain management, 2) adoption of self-management skills (e.g., planning self-monitoring, and attention focus) and targets for change (e.g., exercise level, negative emotions, and dysfunctional thoughts, and 3) the achievement of pain-related outcomes (e.g., increased fitness, decreased suffering, and reduced pain-related interference). Control group was treatment as usual (TAU).	<u>Pain:</u> Brief Pain Inventory<br><br><u>Opioid use:</u><br>Opioid prescription information was collected from the participants medical record and converted to MME	<u>Pain:</u> 24 (14.5%) of 166 E-Health participants achieved a >2 point decrease in pain intensity compared to 13 (6.8%) of 192 TAU participants (odds ratio, 2.4 [95% CL, 1.2-4.9]; *p*=0.02).<br><br><u>Opioid use:</u> 105 (53.6%) of 196 E-Health participants achieved a >15% reduction in opioid use compared with 85 (42.3%) of 201 TAU participants (odds ratio, 1.6 [95% CL, 1.1-2.3]; *p*=0.02).	The majority of participants had a relatively low MME, which may indicate some selection bias. This study is not a double-blinded trial. There was a significant treatment group difference in the month-10 self-assessment completion rates, with higher completion for the TAU (95%) relative to E-Health (83%).
Garland (2024)	RCT	230<br>*	57.5	15.7%	White (85.7%)<br>Black (4.8%)<br>Hispanic/ Latino (3.0%)<br>Native American or Alaskan native (2.6%)<br>Other or missing (3.5%)	Back, osteoarthritis, cervical, neuropathic, fibromyalgia, migraine/ headache, extremity, other	<u>NPI Type:</u> Mindfulness<br><u>Duration:</u> 8 weeks<br><u>Details:</u> MORE intervention provided training in mindfulness, reappraisal, and savoring techniques. Mindfulness included mindful breathing and body scan meditations to attenuate pain and opioid craving. Reappraisal included reframing stress appraisals to reduce catastrophizing and negative emotional reactivity. Savoring included mindfully focusing attention on pleasant events and pleasurable sensations. The active control condition consisted in supportive psychotherapy.	<u>Pain:</u> Brief Pain Inventory<br><br><u>Opioid use:</u><br>Urine drug screens, opioid prescription information was collected from the participants medical record and converted to MME	<u>Pain: The</u> MORE group showed significantly greater reduction in pain outcomes than the control group (*p*=0.025).<br><br><u>Opioid use: The</u> MORE group reduced opioid dose significantly compared to SG (B=0.65, 95% CI=0.07-1.23, *p*=0.029); 20.7% reduction in mean opioid use (18.88mg, SD=8.40mg) for MORE group compared to 3.9% reduction (3.19mg, SD=4.38mg) for control group. MORE group showed significantly greater reduction in opioid dose than the control group (*p*=0.025).	Participants were not blinded to treatment, homogeneous sample of White race male limits generalizability.
Debar (2022)	RCT	850	60.3	67.4%	White (76.6%)<br>Black (12.9%)<br>Other (10.5%)<br>Hispanic (3.3%)	Joint and arthritis, back, generalized, abdominal, neuropathy, fibromyalgia, headache, pelvic,	<u>NPI Type:</u> Cognitive Behavioral Therapy (CBT)<br><u>Duration:</u> 12 weeks<br><u>Details:</u> CBT intervention teaching pain self-management skills in 12 weekly, 90-minute groups delivered by an interdisciplinary team (behaviorist, nurse, physical therapist, and pharmacist) versus usual care.	<u>Pain:</u> Pain intensity and interference with enjoyment of life, general activity, and sleep<br><br><u>Opioid use:</u> Self-reported and	<u>Pain:</u> CBT had larger reductions in pain outcomes at 12-month follow-up compared to usual care (difference, -0.434 point [95% CI, 0.690 to -0.178 point]) and post-treatment (difference, -	The inclusion of only patients with insurance in large integrated health care systems limited generalizability.

						orofacial, other, musculoskelet al chest		converted to MME per 90- day period	0.565 point [CI, -0.796 to - 0.333 point].<br><br>Opioid use: No differences were seen in opioid use at post-treatment (difference, - 2.260 points [CI, -5.509 to 0.989 points]) or at 12-month follow-up (difference, -1.969 points [CI, -6.765 to 2.827 points]).	
Gardiner (2019)	RCT	159	51	86%	Black (56%)<br>Other (36%)<br>White (19%)	Chronic pain (authors did not provide specific types)	NPI Type: Combination NPI<br>Duration: 21 weeks<br>Details: Integrative Medical Group Visits (IMGVs) combines mindfulness techniques, evidence-based integrative medicine, and medical group visits. The IMGVs consisted of a total of 10 in-person medical group visits each lasting 2.5 hours conducted weekly from week 1 to week 9. This was followed by a 12-week maintenance phase where there is access to the technology only (E-Health tool kit). A tenth and final in-person session was conducted at week 21.	Pain: Brief Pain Inventory<br><br>Opioid use: Self-reported	Pain: No differences in pain outcomes at any time point.<br><br>Opioid use: At 21 weeks, the IMGV group reported greater reduction in pain medication use (Odds Ratio: 0.42, CI: 0.18-0.98) compared to controls.	It is possible that 9 weeks of active in-person group visits was not long enough to see a significant change when comparing a routine primary care to medical group visit. The use of self-reported measures. The study did not have the statistical power to conduct a multi-variable regression for reduction in opioids because of the small sample size.
Wartko (2023)	RCT	153	60.7	64%	White (86%)<br>Black (5%)<br>Other (5%)<br>Hispanic (5%)<br>American Indian/ Alaskan Native (5%)<br>Multiracial (3%)<br>Asian (1%)<br>Native Hawaiian/ other Pacific Islander (1%)	Chronic pain (authors did not provide specific types)	NPI Type: Cognitive Behavioral Therapy (CBT)<br>Duration: 18 sessions/1 year<br>Details: CBT-based pain coping skills training intervention with an optional supported opioid taper delivered by telephone in 18 sessions over 1 year by a nurse or physician assistant. The control arm was usual care.	Pain: Pain, Enjoyment of Life, and General Activity<br><br>Opioid use: Self-reported and converted to MME	Pain: No significant differences between intervention and usual care for pain outcomes were found (0.0 [95% CI: -0.5, 0.5], *p*=0.985).<br><br>Opioid use: No significant differences between intervention and usual care for opioid use were found (adjusted mean difference: -2.3 MME; 95% CI: -10.6, 5.9; *p*=0.578).	Generalizability (sample was relatively highly educated, primarily White, and few Hispanic). Excluded participants who could not participant in telephone sessions (e.g., hearing impaired) or were not proficient in understanding, speaking, and reading English.
Groessl (2017)	RCT	150<br><br>*	53.4	26%	White (49.3%)<br>Black (17.3%)<br>Hispanic (20%)<br>Asian/Pacific Islander (6%)<br>Other (6%)<br>Native American (1.3%)	cLBP	NPI Type: Yoga<br>Duration: 12 weeks<br>Details: Yoga intervention consisted of two 60-minute instructor-led yoga sessions per week. The intervention was hatha yoga, consisting of physical yoga postures, movement sequences and regulated breathing. Participants were randomized to either yoga or delayed yoga treatment.	Pain: Brief Pain Inventory<br><br>Opioid use: Self-reported and verified using medical records.	Pain: Differences observed at all three time points (*p*=0.001 for 6 weeks, 0.005 for 12 weeks, 0.013 for 6 months), with larger reductions in pain intensity for yoga participants.<br><br>Opioid use: Significant reduction from 20% to 11% at 12 weeks (*p*=0.007) and 8% after 6 months (*p*<0.001).	Single yoga instructor taught all intervention classes. Attrition was high and the delayed treatment comparison group might be viewed as a less rigorous comparator.
Roseen (2022)	RCT	120<br><br>*	55.5	9.2%	White (89.1%)<br>Black (6.7%)<br>Hispanic (4.2%)	cLBP	NPI Type: Yoga<br>Duration: 12 weeks<br>Details: Yoga intervention consisted of 12 weekly, 75 min yoga classes. Each class included a yoga breathing exercise (Pranayama), discussion of yoga philosophical principles, yoga postures (asanas), and deep relaxation (svasana). Each participant received a copy of *The Back Pain Helpbook*. The control arm was an educational group.	Pain: Defense and Veterans Pain Rating Scale<br><br>Opioid use: Self-reported	Pain: No significant in-between differences were observed for pain.<br><br>Opioid use: No significant in-between differences were observed for opioid use. Post-treatment, fewer yoga than education participants reported	Only half of the participants in the yoga intervention group adhered, which may have biased an effect toward the null. There was a differential loss to follow-up: 10 (16%) in the yoga group and 1 (2%) in education. No report of blinding for participants and those measuring outcomes. Homogeneous sample of White race males limits generalizability.

									pain medication use (55% vs 67%, OR=0.56, 95% CI: 0.26-1.24, *p*=0.15).	
Sandhu (2023)	RCT	608	61	60%	White (96.2%)<br>Black Caribbean (1.0%)<br>Indian (1.0%)<br>Other (0.7%)<br>Black African (0.2%)<br>Black Other (0.2%)<br>Pakistani (0.2%)<br>Prefer not to say (0.2%)	Multisite, cLBP, chronic widespread	<u>NPI Type:</u> Educational Program<br><u>Duration:</u> 3 days intervention and 12 months maintenance<br><u>Details:</u> Group-based educational intervention designed to encourage opioid cessation with a mutually agreeable decision plan between the participant and nurse. The intervention included 3-day-long group meetings held once weekly and led by a trained intervention nurse and by a lay person with chronic nonmalignant pain and experience with opioid tapering. The control arm was usual care.	<u>Pain:</u> Patient-Reported Outcomes Measurement Information System<br><br><u>Opioid use:</u> Self-reported, with a participant report verified in a telephone call from a member of the study team and converted to MME	<u>Pain:</u> No significant between-group differences in pain intensity.<br><br><u>Opioid use:</u> At 12 months, 65 of 225 participants (29%) achieved opioid cessation in the intervention group and 15 of 208 participants (7%) achieved opioid cessation in the usual care group (odds ratio, 5.55 [95% CI, 2.80 to 10.99].	Self-reported measures, participants were not blind to group assignment, study coordinators were regularly unblinded by study participants. Individuals volunteered to participate and were likely more committed to reduce use of opioids than people who did not participate. Only 47% adhered to intervention. Additionally, 33% of participants used a MME of less than 30mg per day at baseline. Results may not be generalized to people using higher MME. Homogeneous sample of White race participants limits generalizability.
Does (2024)	RCT	376	59.8	58.2%	White (67.6%)<br>Hispanic (16.8%)<br>Black (5.3%)<br>Asian (5.1%)<br>Native American (4.3%)	Back, extremity, neck, fibromyalgia, other	<u>NPI Type:</u> Educational Program<br><u>Duration:</u> 4 sessions<br><u>Details:</u> Intervention consisted of four 90 min group sessions conducted by a licensed psychologist with expertise in chronic pain. Groups were intentionally small (3-8 participants) to facilitate discussion and interaction. Each session included an educational presentation and skills practice (e.g., goal setting, guided imagery, patient–provider communication role play, patient portal navigation). The control arm was usual care.	<u>Pain:</u> Patient-Reported Outcomes Measurement Information System<br><br><u>Opioid use:</u><br>Pharmacy dispensation data from the medical record and converted to MME for the 6-month period.	<u>Pain:</u> No significant between-group differences in pain intensity.<br><br><u>Opioid use:</u> A small but not significant decrease in opioid use was found in both groups over the study period. At 12 months, intervention group demonstrated greater medication use (OR=2.72; 95% CI 1.61-4.58).	Study was conducted in an integrated health care system which limits generalizability. Adherence was challenging with 75% of intervention participants completing 3 or more sessions. No report of blinding for participants and those measuring outcomes.
Naylor (2010)	RCT	51	46	86%	White (96%)	Chronic musculoskeletal pain	<u>NPI Type:</u> Digital Technology<br><u>Duration:</u> 4 months<br><u>Details:</u> Therapeutic Interactive Voice Response (TIVR) is an automated, telephone-based tool developed for the maintenance and enhancement of CBT skills. All participants completed group pain-coping skills training (CBT), which consisted of 90-minute weekly sessions over 11 weeks. The experimental group received 4 months of maintenance program via the TIVR. Control group received standard follow-up care.	<u>Pain:</u> Short form of the McGill Pain Questionnaire, the Pain Symptoms Subscale from the Treatment Outcomes in Pain Survey<br><br><u>Opioid use:</u><br>Self-reported	<u>Pain:</u> TIVR showed significant improvement at 8-month follow-up for pain scores (*p*<0.0001), compared to the control group.<br><br><u>Opioid use:</u> Opioid use reduced in the TIVR group in both follow-ups: 4- and 8-months post-CBT. At 8-month follow-up, 21% of the TIVR participants stopped using opioids. There were significant between-group differences in opioid use at 8-month follow-up (*p*=0.004).	Small sample size; only 29 out of the 51 participants were using opioids, which resulted in fewer subjects for the medication effect analyses. No report of blinding for participants and those measuring outcomes. Homogeneous sample of White race participants limits generalizability.
Nielssen (2019)	RCT	471	50	79.6%	NA	Arthritis, back, fibromyalgia, other	<u>NPI Type:</u> Educational Program<br><u>Duration:</u> 8 weeks<br><u>Details:</u> The pain online educational course consisted of a transdiagnostic psychological intervention and provided therapeutic information and taught self-management skills to manage pain and psychological difficulties. This course provided information about opioid medications. The study included a wait-list control group.	<u>Pain:</u> Roland–Morris Disability Questionnaire, Wisconsin Brief Pain Questionnaire<br><br><u>Opioid use:</u> Self-reported and converted to MME	<u>Pain:</u> Significantly larger reduction in pain outcomes with the intervention group compared to the control group.<br><br><u>Opioid use:</u> Significant reduction in opioid use compared to control group.	Self-reported measures, population characteristics regarding race were missing, details regarding power analyses were not included.

Day<br>(2019)	Pilot RCT	69	50.7	52%	White (89%)<br>Other (7%)<br>Asian (4%)	cLBP	<u>NPI Type:</u> Combination NPI<br><u>Duration:</u> 8 weeks<br><u>Details:</u> Group-delivered Cognitive Therapy (CT), Mindfulness Meditation (MM), and Mindfulness-based Cognitive Therapy (MBCT) for cLBP. Participants were randomized to one of the treatments (CT, MM, or MBCT), which consisted of eight weekly 2-hour sessions.	<u>Pain:</u> Numeric rating scale<br><br><u>Opioid use:</u><br>Self-reported opioid use in the past week	<u>Pain:</u> Post-treatment, the intent-to-treat group showed significant improvements for pain intensity (*p*<0.001), with no significant between group differences.<br><br><u>Opioid use:</u> For the intent-to-treat group, there was no significant difference (*p*=0.549) in opioid use between pre-treatment (48%) and post-treatment (43%). Opioid use decreased significantly (p=0.012) from pre-treatment (49%) to 3-month follow-up (28%), but opioid use at post-treatment (40%) and 6-month follow-up (33%) was not significantly reduced (*p*=0.289) compared with pre-treatment.	Generalizability (sample was comprised primarily of White race, well-educated individuals). The pilot nature of the study was not sufficiently powered to detect small effect sizes and type 1 errors could be inflated. The study did not include a nonactive control group. All instruments were delivered by one therapist—which might introduce a degree of bias.
Spangeus<br>(2023)	Pilot RCT	21	72	95.2%	NA	Spinal osteoporosis	<u>NPI Type:</u> Educational Program<br><u>Duration:</u> 10 weeks<br><u>Details:</u> The three intervention arms: theory only [T-group], theory and physical exercise [Tph group], and theory and mindfulness/medical yoga [TMMY group] included the same theoretical lectures organized as a 1 hr weekly theory session for 10 weeks. In addition, the TPh and TMMY groups had a 1 hr training session scheduled in adjunction to theory sessions.	<u>Pain:</u> Numeric Pain Scale<br><br><u>Opioid use:</u> Self-reported opioid use	<u>Pain:</u> Significant improvements post-treatment on pain outcomes were found.<br><br><u>Opioid use:</u> Significant reduction in opioid use (25%) at baseline and (14%) at post-treatment were found.	The pilot nature of the study was not sufficiently powered, small sample size, population characteristics regarding race were missing, and self-reported measures.
Nelli<br>(2023)	Pilot RCT	45	57	91.2%	White (70%)<br>Black (30%)	Fibromyalgia	<u>NPI Type:</u> Device<br><u>Duration:</u> 2 weeks<br><u>Details:</u> The three visual light spectrum-based intervention arms: clear eyeglasses (control), green eyeglasses, or blue eyeglasses. Patients were instructed to wear their study eyeglasses for at least 4 hours per day for 2 weeks while awake.	<u>Pain:</u> Numeric Scale<br><br><u>Opioid use:</u> Self-reported and converted to MME	<u>Pain:</u> The reduction in pain scores was 67%, 50%, and 45% for the green, blue, and clear glasses groups (*p*=0.56). No significant differences in pain score reduction between groups were found.<br><br><u>Opioid use:</u> Greater than 10% reduction in opioid use was achieved and found 33%, 11%, and 8% of the green, blue, and clear eyeglasses groups (*p*=0.23).	The pilot nature of the study was not sufficiently powered to detect opioid effects, small sample size, high attrition rate, short duration of 2 weeks for the intervention.
Moffat<br>(2023)	Observational	13968<br><br>*	NA	42%	NA	CNCP<br>(unspecified)	<u>NPI Type:</u> Combination NPI<br><u>Duration:</u> 22 months<br><u>Details:</u> National intervention that included audit and feedback, deprescribing guidance, information on catastrophizing assessment, pain neuroscience education, and a cognitive tool for use by patients with their healthcare providers.	<u>Pain:</u> NA<br><br><u>Opioid use:</u> Identified using the Australian Pharmaceutical Benefits Scheme item number and converted to MME	<u>Pain:</u> NA<br><br><u>Opioid use:</u> Calculated change in predicted trends with and without the intervention 25387 (95% CI 24676, 26131).	Inability to determine causation, single-arm time series design without comparison. A multi-strategic approach makes it difficult to identify which intervention had the most impact and population characteristics regarding race and pain type were missing.
Zeliadt<br>(2022)	Observational	4869<br><br>*	NA	17.8%	Black (23%)<br>White (70%)<br>Other (3%)<br>Not reported (4%)	Multiple, back, extremity, chest, neck, other	<u>NPI Type:</u> Combination NPI<br><u>Duration:</u> 18 months<br><u>Details:</u> Veterans participated in Whole Health and complementary and integrative health therapies (CIH) through a variety of referral and recruitment methods across the 18 pilot sites. Whole Health	<u>Pain:</u> NA<br><br><u>Opioid use:</u> Extracted from VA's pharmacy managerial cost accounting national	<u>Pain:</u> NA<br><br><u>Opioid use:</u> Opioid use decreased by -12% in one year among veterans who began CIH compared to	CIH therapies and Whole Health services were not randomized; there is the potential for selection bias. No measure of pain outcomes.

							involves referral to CIH therapies, including the following: acupuncture, chiropractic care, therapeutic massage, yoga, tai chi/qigong, meditation, guided imagery, clinical hypnosis, and biofeedback. Whole Health services included Whole Health pathway, Whole Health education/skills classes, personal health planning, and Whole Health coaching. Patients exposed to Whole Health services were offered nine CIH alone or in combination with Whole Health services.	data and converted to MME.	similar veterans who used conventional care; -4.4% among veterans who used only Whole Health services compared to conventional care, and -8.5% among veterans who used both CIH combined with Whole Health services compared to conventional care.	
Huffman (2019)	Observational	1681	46.6	65.3%	White (83.2%)<br>Black (10.5%)<br>Other (3.2%)<br>Missing (3.1%)	cLBP, headache, neck, fibromyalgia, joint	NPI Type: Combination NPI<br>Duration: 4 weeks<br>Details: Interdisciplinary CNCP rehabilitation program (including medication management, individual and group psychotherapy, cognitive behavioral group, interventions, psychoeducation, physical and occupational therapy, substance use education, weaning from habituating medications, and optional free monthly after-care). The program was delivered for 3-4 weeks, 9.5 hours, 5 days per week.	Pain: Numeric Rating Scale<br><br>Opioid use: Self-reported	Pain: Pain on discharge, at 6 months, and at 12 months was significantly lower compared to on admission (*p*<0.05).<br><br>Opioid use: There were significantly fewer patients using opioids *p*<0.05) post-treatment. At 6-month follow-up, 76.3% maintained opioid cessation, 14.6% resumed opioid use, 5.8% continued to use opioids, and 3.4% discontinued opioid use. At 12-month follow-up, 14.6% maintained opioid cessation, 5.8% resumed opioids, 3.4% continued to use opioids, and 76.3% discontinued opioid use.	Missing longitudinal data; 40.5% of study participants did not return for follow-up surveys.
Townsend (2008)	Observational	373	44.5	79.1%	White (95.7%)<br>Other (4.3%)	cLBP, fibromyalgia, headache, generalized, abdominal, neck, other	NPI Type: Combination NPI<br>Duration: 3 weeks<br>Details: Mayo Clinic Comprehensive Pain Rehabilitation Center (MCPRC) is an intensive outpatient interdisciplinary pain rehabilitation program for patients with CNCP to improve adaptation to pain and related symptoms. A CBT model serves as the basis for treatment at the MCPRC and incorporates physical therapy, occupational therapy, biofeedback and relaxation training, stress management, wellness education (e.g., sleep hygiene, healthy diet), and pain management training (e.g., activity moderation, elimination of pain behaviors).	Pain: Multidimensional Pain Inventory<br><br>Opioid use: Verified using medical records and converted to MME	Pain: Significant improvement was found in pain outcomes post-treatment (*p*< 0.001) and six-month post-treatment (*p*< 0.001).<br><br>Opioid use: At discharge, 176 (92.6%) of the opioid group had completed the taper of opioids (x^2 =20.57; df=1, *p*<0.001).	No control group. The applicability of these findings to other CNCP populations may be limited because of inherent selection bias in the study sample and attrition is another possible source of bias in drawing conclusions from the results. Homogeneous sample of White race participants limits generalizability.
Ward (2022)	Observational	237<br><br>*	57.1	30.4%	Black (78.2%)<br>White (17.8%)<br>Hispanic (1.6%)<br>Other (1.2%)<br>Missing (1.2%)	Chronic Pain (unspecified)	NPI Type: Combination NPI<br>Duration: 10 weeks<br>Details: Empower Veterans Program (EVP) , an interdisciplinary pain rehabilitation and functional restoration program option for functional restoration for high-impact chronic pain, offered in a large metro-area Veterans Health Administration (VA) system. Veterans participated in closed cohorts that met once a week in three integrated sessions for 10 weeks: 1) EVP Acceptance and Commitment Therapy, 2) EVP Whole Health with motivational interviewing and informed discussions of self-care and mindfulness training, 3) EVP Mindful Movement with body awareness and whole-body movement training.	Pain: Pain Numeric Scale<br><br>Opioid use: Number of days with prescription opioids determined by VA pharmacy data	Pain: No significant improvement to pain scores noted.<br><br>Opioid use: No significant differences in percentage of opioid use found one year pre-post treatment for both EVP-engaged and not-engaged participants.	This quality improvement evaluation is not designed to produce results that could be generalized. The retrospective, observational design is limited in producing causal conclusions. This study is likely to be biased to an unknown degree by unmeasured confounding factors.

Van Der Merwe (2021)	Observational	164 *	45.3	10.1%	NA	Spinal, extremity, face, abdominal	NPI Type: Combination NPI<br>Duration: 10 days<br>Details: A chronic pain management program (PMP) was delivered for 10 days, for a total of 60 hours by a team of clinicians: clinical psychologist, physiotherapist, nurse, medical consultant, and psychiatrist. PMP includes CBT (mindfulness-based CBT for pain and compassion-focused therapy), medication rationalization, ensuring recommended daily doses are not exceeded, and reducing dose or discontinuing medication.	Pain: Brief Pain Inventory<br><br>Opioid use: Self-reported	Pain: Significant improvement with treatment ($p< 0.001$).<br><br>Opioid use: Approximately 25% stopped opioid use and 17% had reduced opioid use post-treatment.	There was no follow-up beyond the last day of the program, so no evidence can be provided for maintenance of treatment gains in the longer term. Missing values were a problem for some scales, particularly the IES-6. Population characteristics regarding race were missing.
Hooten (2007)	Observational	159	45	86.2%	NA	Fibromyalgia	NPI Type: Combination NPI<br>Duration: 3 weeks<br>Details: Outpatient pain rehabilitation program incorporating physical reconditioning, biofeedback and relaxation training, stress management, chemical health education, activity moderation, and elimination of pain behaviors. Patients attended 8 hours daily for 15 consecutive working days.	Pain: Multidimensional Pain Inventory<br><br>Opioid use: Medical chart review	Pain: Significant improvement with program treatment ($p< 0.001$).<br><br>Opioid use: Compared with admission, opioid use at post-treatment was significantly reduced ($p< 0.001$).	No comparison or control group and population characteristics regarding race were missing.
Davis (2018)	Observational	156	NA<br><br>age range 18-70+	71%	NA	Back, joint, neck, headache, abdominal, extremity, carpal tunnel, hip, jaw	NPI Type: Acupuncture<br>Duration: 12 sessions/60 days<br>Details: 12 total acupuncture treatments within 60-day period. Acupuncture type was determined by each acupuncturist as appropriate for each patient, and treatment permitted inclusion of electroacupuncture, infrared, moxibustion, manual therapy, cupping, gua sha, topical herbal liniments, and Chinese herbal medicines.	Pain: Patient-Reported Outcomes Measurement Information System<br><br>Opioid use: Self-reported	Pain: Significant improvements in pain intensity ($p<0.01$).<br><br>Opioid use: Approximately 32% of patients using opioids reported reductions in use post-intervention.	No placebo or sham acupuncture comparison group; consequently, unable to conclude that changes observed in patients were due to acupuncture specifically. Population characteristics regarding race were missing.
Schumann (2020)	Observational	134	71	38.1%	White (95.5%)<br>Other (4.5%)	cLBP, generalized, fibromyalgia, other	NPI Type: Combination NPI<br>Duration: 3 weeks<br>Details: 3-week IPRP with physician-supervised medication tapering, occupational therapy, and a cognitive behavioral therapy model serves as the basis for treatment.	Pain: West Haven Yale Multidisciplinary Pain Inventory<br><br>Opioid use: Self-reported and converted to MME	Pain: Significant treatment effects ($p<0.001$) with large effect sizes were observed.<br><br>Opioid use: Significant reductions ($p<0.01$) in opioids were found post-treatment. All participants in the opioid group completed the opioid taper and discontinued use.	Homogeneous sample of White race participants limits generalizability and no control group for follow-up comparisons.
Gibson (2020)	Observational	99 *	60	16.2%	White (61.6%)<br>Black (15.2%)<br>Hispanic or Latino (9.1%)<br>Other (14.1%)	Chronic pain (unspecified)	NPI Type: Combination NPI<br>Duration: 3 months<br>Details: Biopsychosocial integrative pain team (IPT) model includes personalized care plans established for each patient's needs with aim of reducing opioid risk and chronic pain. IPT (e.g., chiropractic treatment, cognitive behavioral therapy, massage, meditation, yoga, hydrotherapy, physical therapy, breathing, relaxation) involves in-person or telehealth care by co-located medical provider, psychologist, and pharmacist.	Pain: Brief Pain Inventory<br><br>Opioid use: Self-reported	Pain: No significant change in pain severity (p=0.11, ES=0.16).<br><br>Opioid use: At baseline, 77 participants were prescribed long-term opioids, 6 (7%) discontinued between baseline and follow-up.	Self-reported measures, no control, homogeneous sample of mostly men limits generalizability.
Van Hooff (2012)	Observational	85	42.9	59%	NA	cLBP	NPI Type: Combination NPI<br>Duration: 10 days<br>Details: Residential program with follow-up at 1-month and 1-year post-treatment. The program consists of 100 hours of participant contact time, approximately 50 hours of cognitive behavioral training, 35 hours of graded physical activities, and 15 hours of education in which the cognitive behavioral principles are integrated. Team consists of psychologist, physiotherapist, and occupational therapist.	Pain: Visual Analogue Scale<br><br>Opioid use: Self-reported	Pain: No significant improvement at 1-year follow-up (p=0.34).<br><br>Opioids use: Minimal reduction was found, 25% of patients used opioids (15% weak opioid, 10% strong opioid) at pre-treatment, and 14% of patients used opioids (11% weak	Due to the intervention including a wide range of techniques based on principles of CBT, it is unclear which techniques, or which parts of the intervention, are responsible for the observed effect. Self-reported measures; study does not show data for immediate post-treatment or 1-month post-treatment, and population characteristics regarding race were missing.

									opioid, 3% strong opioid) at 2-year follow-up.	
Gilliam (2020)	Observational	762	48.5	70.2%	White (90.6%)<br>Other (9.4%)	Fibromyalgia, cLBP, generalized, headache, other	NPI Type: Combination NPI<br>Duration: 15 days<br>Details: Mayo Clinic Pain Rehabilitation Center is an intensive, outpatient interdisciplinary rehabilitation program focusing on functional restoration. The program includes 15 days in duration (8 hours daily for 15 consecutive working days) and combines functional restoration (occupational therapy) with cognitive behavioral therapy as its chief components.	Pain: West Haven Yale Multidimensional Pain Inventory<br><br>Opioid use: Medical records, medicine bottles, patient report, and state prescription monitoring programs and converted to MME	Pain: Significant improvements were found for pain outcomes.<br><br>Opioid use: Significant improvements were found for opioid use. At discharge, all patients (31.8%, *n*=242) taking opioids at pre-treatment had completed the taper and discontinued opioid use.	No randomization to control condition, self-report measures, and homogeneous sample of White race participants limits generalizability.
Trinh (2023)	Observational	74	54.4	51.3%	White (82.4%)<br>Black (14.9%)<br>Hispanic (1.4%)<br>Asian (1.4%)	cLBP, neck, extremity, shoulder, hip, chest	NPI Type: Device<br>Duration: 30 days<br>Details: H-Wave device stimulation is a type of transcutaneous electrotherapy that uses a specific proprietary "H waveform" (biphasic, exponentially decaying, low frequency, long pulse duration) to stimulate muscle fiber contractions, which are non-fatiguing and low-tension, mimicking natural voluntary motor contractions. This leads to increased blood flow via nitric oxide-dependent vasodilation, angiogenesis (formation of new blood vessels), resolution of edema, and anesthesia (in high frequency mode).	Pain: Brief Pain Inventory, Visual Analogue Scale<br><br>Opioid use: self-reported, compensation claimants	Pain: Significant reduction in pain post-H-Wave treatment (p<0.0001)<br><br>Opioid use: Approximately 49% of the patients taking opioids prior to the H-Wave device intervention subsequently reduced or stopped their usage.	No control or comparison group; self-reported measures.
Passmore (2022)	Observational	62	48.6	61.3%	White (48.4%)<br>Metis (24.2%)<br>First Nations (24.2%)<br>Black (1.61%)<br>Latino (1.61%)	Cervical, thoracic, lumbar, sacral, extremity	NPI Type: Chiropractic<br>Duration: NA<br>Details: Chiropractic treatment consisted in diversified spinal or extremity joint manipulation and/or mobilization, soft tissue therapy, acupuncture, and/or other modalities, including but not limited to ultrasound, electrical stimulation, thermal treatment, exercise, home advice, and Kinesiotaping, when clinically indicated. Treatments were delivered by a licensed chiropractor. New patient assessments were scheduled for 30 to 60 minutes, while treatment visits and re-evaluation visits were 15 to 30 minutes.	Pain: Numeric Rating Scale<br><br>Opioid use: self-reported	Pain: Significant decrease in pain intensity was found.<br><br>Opioid use: Significant reduction of opioid use was found (p=0.012); approximately 59.0% reduction post-treatment.	A limitation of the dataset is that the number of weeks in a course of care is not possible to determine when multiple courses of care are engaged in by a single patient. No control group or randomization, and unspecified duration of care to discharge.
Buchfuhrer (2023)	Observational	20	62.9	40%	White (90%)<br>Hispanic (10%)	Refractory restless legs syndrome (neurological)	NPI Type: Device<br>Duration: 21 days<br>Details: Tonic motor activation (TOMAC) is a nonpharmacological therapeutic device indicated for refractory restless legs syndrome and includes two therapy units worn bilaterally on the lower legs, which produce a current controlled, charged balance 40000 Hz stimulation intensity >40 milliamps. TOMAC therapy units are placed over the peroneal nerve at the head of the fibula bone.	Pain: Clinician Global Impression of Improvement<br><br>Opioid use: Self-reported and converted to MME	Pain: No changes to restless legs syndrome severity found.<br><br>Opioid use: Approximately 70% of participants (14/20) successfully reduced opioid use >20%; 29.9% mean opioid reduction (*SD*=23.7%, *n*=20) from 39.0 to 26.8 MME per day post-TOMAC treatment.	Small sample size, open-label, single-arm design, and short duration. Possible placebo effect and bias in patient assessments. Homogeneous sample of White race participants limits generalizability.
Barrett (2021)	Observational	17	54	70.6%	White (82.4%)<br>Black (5.9%)<br>Multiracial (11.8%)	Back, arthritis, fibromyalgia, neck, neuropathy, headache, complex regional pain syndrome, pelvic, other	NPI Type: Combination NPI<br>Duration: 8 weeks<br>Details: Dialectical pain management (DPM), a hybrid skills-based group intervention. 8 weekly group sessions, 1.75 hours each, 6-9 participants in each group. DPM incorporates CBT and acceptance and commitment therapy (ACT).	Pain: Brief Pain Inventory<br><br>Opioid use: Self-reported and converted to MME	Pain: No significant changes in pain severity (5.9 vs. 5.93, p=0.913).<br><br>Opioid use: Five participants (38.5%) reported decreasing their opioid use since baseline. Of these five, opioid use reductions were 17%,	Single-site, single-arm feasibility trial without a control group, skewed gender and racial composition, and medication dosages and changes in dose were not verified by medical records or state monitoring databases.

									25%, 34%, 55%, and 74%. The mean opioid use decreased from 138.17 mg (*SD* = 83.99) to 101.21 mg (*SD* = 45.71).	
Matyac (2022)	Observational	13	NA	62%	NA	cLBP, neuropathic, joint, fibromyalgia, other	<u>NPI Type:</u> Educational Program<br><u>Duration:</u> 5 weeks<br><u>Details:</u> The program included 3 on-site, in-person group education sessions (neurophysiology, biopsychosocial, risks and benefits of opioids, non-opioid alternatives, and pain management techniques) lasting 2 hours each and jointly delivered by a physical therapist, a behaviorist, and a pharmacist. The 3 educational sessions were scheduled over a five-week period with a one-week gap between each.	<u>Pain:</u> Pain, Enjoyment, and General Activity<br><br><u>Opioid use:</u> Self-reported and converted to MME	<u>Pain:</u> The program was associated with decreased pain intensity.<br><br><u>Opioid use:</u> Although not significant, the program was associated with reduced opioid use.	Small sample size (no statistically significant differences could be determined), single-arm quasi-experimental design (no control or randomization), self-report measures, and population characteristics regarding race and mean age were missing.
Nilsen (2010)	Observational	11	43	81.8%	NA	CNCP (Unspecified)	<u>NPI Type:</u> Cognitive Behavioral Therapy<br><u>Duration:</u> 8 weeks<br><u>Details:</u> Six 1-hour sessions of CBT during an 8-week period by two CBT specifically-trained physicians.	<u>Pain:</u> Brief Pain Inventory<br><br><u>Opioid use:</u> Codeine (milligram) use and blood sample taken at the first session for genetic polymorphism CTP2D6	<u>Pain:</u> No significant changes (p>0.05) were found to mid-treatment (d = 0.3), post-treatment (d = 0.4), or to follow-up (d = 0.4).<br><br><u>Opioid use:</u> A significant decrease in codeine use was found from pre- to mid-treatment (t = 11.4, p<0.001; d = 2.2), pre- to post-treatment (t = 11.8, p<0.001; d = 2.9), pre-treatment to follow-up (t = 11.7, p<0.001; d = 2.9), and from mid- to post-treatment (t = 6.1, p<0.001; d = 1.4).	Small sample size without any control group. Neither ratings of competence nor adherence were systematically collected and calculated. Population characteristics regarding race were missing.
McCrae (2020)	Secondary Analysis	113	54	85.8%	White (79.6%)<br>Black (17.7%)<br>Native Indian/Alaskan Native (1.8%)<br>Biracial (0.9%)	Fibromyalgia	<u>NPI Type:</u> Cognitive Behavioral Therapy<br><u>Duration:</u> 8 weeks<br><u>Details:</u> 8 weekly 50 min sessions delivered individually in-person by doctoral clinical psychology students. Sessions for pain included pain education, progressive muscle relaxation activity rest cycle, activity pacing and autogenic relaxation, problem solving and visual imagery, cognitive restructuring, and maintenance.	<u>Pain:</u> NA<br><br><u>Opioid use:</u> Self-reported	<u>Pain:</u> NA<br><br><u>Opioid use:</u> There were no significant effects for frequency of opioid use between groups (CBT-insomnia, CBT-pain, waitlist control).	Focus on individuals with fibromyalgia; it is unclear whether these findings generalize to other chronic pain conditions. Homogeneous sample of White race, female, middle-aged to older participants limits generalizability.
Miller-Matero (2022)	Secondary Analysis	60	63.3	100%	Black (88.3%)<br>White (11.7%)	Chronic Musculoskeletal pain condition	<u>NPI Type:</u> Combination NPI<br><u>Duration:</u> 5 sessions<br><u>Details:</u> The 5 psychological intervention sessions were approximately 30-45 minutes each and were composed of evidence-based strategies for chronic pain (CBT, mindfulness, and acceptance-based strategies)	<u>Pain:</u><br>Brief Pain Inventory<br><br><u>Opioid use:</u> EHRs verified and converted to MME	<u>Pain:</u> Intervention significantly reduced pain outcomes (p=0.048).<br><u>Opioid use:</u> Though not significant, the intervention showed lower odds of having an opioid prescription 6-months post-intervention (p=0.09, OR=0.32).	The study was not appropriately powered to detect differences between the intervention and control groups for opioid use after the intervention. Homogeneous sample of female, middle-aged to older participants limits generalizability.<br><br>*Mean age, race, pain scales, and additional measures were obtained from original RCT [74]*

Abbreviations: nonpharmacological intervention (NPI), chronic noncancer pain (CNCP), chronic low back pain (cLBP), cognitive behavioral therapy (CBT), cognitive therapy (CT), mindfulness meditation (MM), mindfulness-based cognitive therapy (MBCT), electronic medical record (EHR), Veterans Health Administration (VA), Biopsychosocial Integrated Pain Team (IPT), Brief Pain Inventory (BPI), Pain Catastrophizing Scale (PCS), Current Opioid Misuse Measure (COMM), Pain Treatment Satisfaction Scale (PTSS), randomized controlled trial (RCT), Patient Health Questionnaire (PHQ-8 and PHQ-9), Generalized Anxiety Disorder Scale (GAD-7), Initiative on Methods, Measurement, and Pain Assessment in Clinical Trials (IMMPACT), participant global satisfaction with treatment (PGATS), Patient-Reported Outcomes Measurement Information System (PROMIS), interdisciplinary pain rehabilitation program (IPRP), morphine milligram equivalent (MME), West Haven Yale Multidimensional Pain Inventory (WHYMPI), Center for Epidemiologic Studies Depression Scale (CES-D), Defense and Veterans Pain Rating Scale (DVPRS), Chronic Pain Acceptance Questionnaire (CPAQ-8), Roland–Morris Disability Questionnaire (RMDQ), System Usability Scale (SUS), Multidimensional Pain Inventory (MPI), Coping Strategies Questionnaire—Catastrophizing subscale (CSQ-C), Short Form-36 Health Status Questionnaire (SF-36), Nondual Awareness Dimensional Assessment (NADA-state), Perceived Body Boundaries Scale (PBBS), Pain Disability Index (PDI), Clinician Global Impression of Improvement (CGI-I), Pain Self-Efficacy Scale (PSEQ), Multidimensional Pain Inventory (MPI), Patient Activation Measure (PAM-13), standard deviation (*SD*), Short Opioid Withdrawal Scale (SHOWS), tonic motor activation (TOMAC), acceptance and commitment therapy (ACT), dialectical pain management (DPM), pain management program (PMP), supportive group (SG), Integrative Medical Group Visits (IMGVs), Mayo Clinic Comprehensive Pain Rehabilitation Center (MCPRC), Clinician Global Impression of Improvement (CGI-I), Empower Veterans Program (EVP), complementary and integrative health therapies (CIH), Therapeutic Interactive Voice Response (TIVR), treatment as usual (TAU), mindfulness-oriented recovery enhancement (MORE), opioid risk (ORT), Stratification Tool for Opioid Risk Mitigation (STORM), Impact of

Events Scale (IES-6), Beck Depression Inventory (BDI), Drug Misuse Index (DMI), Post Traumatic Stress Symptoms (PCL-C), Fall Risk and Physical Activity (FES-I), European Quality of Life (EQ-5D-3L and RAND-36), Quality of Life Questionnaire in the European Foundation for Osteoporosis (Qualeffo-41), Pain, Enjoyment, and General Activity (PEG), hospital anxiety and depression scale (HADS); *veteran or active duty participants.

Table S2. Methodological Quality Assessment

**First Arthur Name and Year**		Garcia (2021)	Jensen (2020)	Zheng (2019)	Garland (2022)	Hudak (2021)	Wilson (2023)	Garland (2024)	DeBar (2022)	Gardiner (2019)	Wartko (2023)	Groessl (2017)	Roseen (2022)	Sandhu (2023)	Does (2024)	Naylor (2010)	Nielssen (2019)	Day (2019)	Spangeus (2023)	Nelli (2023)	Moffat (2023)
Reporting																					
1	Is the hypothesis/aim/objective of the study clearly described? (1 = yes, 0 = no)	1	1	1	1	1	1	1	1	1	1	0	1	1	0	1	1	1	1	1	1
2	Are the main outcomes to be measured clearly described in the introduction or method section? (1= yes, 0 = no)	1	1	1	1	1	1	1	1	1	1	1	1	1	1	1	1	1	1	1	1
3	Are the characteristics of the patients included in the study clearly described? (1 = yes, 0 = no)	1	1	1	1	1	1	1	1	0	0	1	1	1	1	1	0	1	0	1	0
4	Are the interventions of interest clearly described? (1 = yes, 0 = no)	1	1	1	1	1	1	1	1	1	1	1	1	1	1	1	1	1	1	1	1
5	Are the distributions of principal	2	2	2	2	2	2	2	2	2	2	2	2	2	2	2	2	2	2	2	2

	confounders in each group of subjects to be compared clearly described? (2 = yes, 1 = partially, 0 = no)																				
6	Are the main findings of the study clearly described? (1 = yes, 0 = no)	1	1	1	1	1	1	1	1	1	1	1	1	1	1	1	1	1	1	1	1
7	Does the study provide estimates of the random variability in the data for the main outcomes? (1 = yes, 0 = no)	1	1	1	1	1	1	1	1	1	1	1	1	1	1	1	1	1	1	1	1
8	Have all important adverse events that may be a consequence of the intervention been reported? (1 = yes, 0 = no)	1	1	1	1	1	1	1	1	1	1	1	1	1	1	1	1	1	1	1	1
9	Have the characteristics of patients lost to follow-up been described? (1 = yes, 0 = no)	1	1	1	1	1	1	1	1	1	1	1	1	1	1	1	1	1	1	1	1
10	Have the actual probability values been	1	1	1	1	1	1	1	1	1	1	1	1	1	1	1	1	1	1	1	1

	reported (e.g., 0.035 rather than <0.05) for the main outcomes except where the probability value is less than 0.001? (1 = yes, 0 = no)																				
Reporting Subtotal (score range, 0-11)		11	11	11	11	11	11	11	11	10	10	10	11	11	10	11	10	11	10	11	10
External Validity																					
11	Were the subjects asked to participate in the study representative of the entire population from which they were recruited? (1 = yes, 0 = no)	1	1	1	1	1	1	1	1	1	1	1	1	1	1	1	1	1	1	1	1
12	Were those subjects who were prepared to participate representative of the entire population from which they were recruited? (1 = yes, 0 = no)	1	1	1	1	1	1	1	1	1	1	1	1	0	0	0	0	0	0	1	0
13	Were the staff, places, and facilities	1	1	1	1	1	1	1	1	1	1	1	1	1	1	1	1	1	1	1	1

	where the patients were treated representative of the treatment the majority of patients receive? (1 = yes, 0 = no)																				
External Validity Subtotal (score range, 0-3)		3	3	3	3	3	3	3	3	3	3	3	3	2	2	2	2	2	2	3	2
Internal Validity - Bias																					
14	Was an attempt made to blind study subjects to the intervention they have received? (1 = yes, 0 = no)	1	1	1	0	0	0	0	0	0	0	0	0	0	0	0	0	0	0	0	0
Author First Name and Year		Garcia (2021)	Jensen (2020)	Zheng (2019)	Garland (2022)	Hudak (2021)	Wilson (2023)	Garland (2024)	DeBar (2022)	Gardiner (2019)	Wartko (2023)	Groessl (2017)	Roseen (2022)	Sandhu (2023)	Does (2024)	Naylor (2010)	Nielsse n (2019)	Day (2019)	Spangeus 2023)	Nelli (2023)	Moffat (2023)
15	Was an attempt made to blind those measuring the main outcomes of the intervention? (1 = yes, 0 = no)	1	1	1	1	1	1	1	1	1	1	1	0	0	0	0	0	1	1	0	0
16	If any of the results of the study were based on "data dredging", was this made clear? (1 = yes, 0 = no)	1	1	1	1	1	1	1	1	1	1	1	1	1	1	1	1	1	1	1	1
17	In trials and cohort	1	1	1	1	1	1	1	1	1	1	1	1	1	1	1	1	1	1	1	1

	studies, do the analyses adjust for different lengths of follow-up of patients, or in case–control studies, is the time period between the intervention and outcome the same for cases and controls? (1 = yes, 0 = no)																				
18	Were the statistical tests used to assess the main outcomes appropriate? (1 = yes, 0 = no)	1	1	1	1	1	1	1	1	1	1	1	1	1	1	1	1	1	1	1	1
19	Was compliance with the intervention(s) reliable? (1 = yes, 0 = no)	1	1	1	1	1	1	1	1	1	1	1	1	1	1	1	1	1	1	1	1
20	Were the main outcome measures used accurate (valid and reliable)? (1 = yes, 0 = no)	1	1	1	1	1	1	1	1	1	1	1	1	1	1	1	1	1	1	1	1
Internal Validity - Bias Subtotal (score range, 0-7)		7	7	7	6	6	6	6	6	6	6	6	5	5	5	5	5	6	6	5	5
Internal Validity - Confounding																					
21	Were the patients	1	1	1	1	1	1	1	1	1	1	1	1	1	1	1	1	1	1	1	1

	in different intervention groups (trials and cohort studies) or were the cases and controls (case-control studies) recruited from the same populati on? (1 = yes, 0 = no)																				
22	Were study subjects in different intervention groups (trials and cohort studies) or were the cases and controls (case–control studies) recruited over the same time period? (1 = yes, 0 = no)	1	1	1	1	1	1	1	1	1	1	1	1	1	1	1	1	1	1	1	1
23	Were the study subjects randomized to intervention groups? (1 = yes, 0 = no)	1	1	1	1	1	1	1	1	1	1	1	1	1	1	1	1	1	1	1	0
24	Was the randomized intervention assignment concealed from both	1	1	1	0	0	0	0	0	0	0	0	0	0	0	0	0	0	0	0	0

	patients and health care staff until recruitment was complete and irrevocable? (1 = yes, 0 = no)																				
25	Was there adequate adjustment for confounding in the analyses from which the main findings were drawn? (1 = yes, 0 = no)	1	1	1	1	1	1	1	1	1	1	1	1	1	1	1	1	1	1	1	1
26	Were losses of patients to follow-up taken into account? (1 = yes, 0 = no)	1	1	1	1	1	1	1	1	1	1	1	1	1	1	1	1	1	1	1	1
	Internal Validity - Confounding Subtotal (score range, 0-6)	6	6	6	5	5	5	5	5	5	5	5	5	5	5	5	5	5	5	5	4
Power																					
27	Did the study mention having conducted a power analysis to determine the sample size needed to detect a significant	2	2	2	2	2	2	2	2	2	2	2	2	2	2	1	0	0	0	0	0

	difference in effect size for one or more outcome measure? (2 = yes, ≥2 outcomes, 1 = yes, 1 outcome, 0 = no)																				
Author First Name and Year		Garcia (2021)	Jensen (2020)	Zheng (2019)	Garland (2022)	Hudak (2021)	Wilson (2023)	Garland (2024)	DeBar (2022)	Gardiner (2029)	Wartko (2023)	Groessl (2017)	Roseen (2022)	Sandhu (2023)	Does (2024)	Naylor (2010)	Nielssen (2019)	Day (2019)	Spangeus 2023)	Nelli (2023)	Moffat (2023)
Power Subtotal (score range, 0-2)		2	2	2	2	2	2	2	2	2	2	2	2	2	2	1	0	0	0	0	0
Total Quality Index Score (range, 0-29 points)		29	29	29	27	27	27	27	27	26	26	26	26	25	24	24	22	24	23	24	21

Table S2. (continued)

**Author First Name and Year**		Zeliadt (2022)	Huffman (2019)	Townsend (2008)	Ward (2022)	Van Der Merwe (2020)	Hooten (2007)	Davis (2018)	Schumann (2020)	Gibson (2020)	Van Hooff (2012)	Gilliam (2020)	Trinh (2023)	Passmore (2022)	Buchfuhrer (2023)	Barrett (2021)	Matyac (2022)	Nilsen (2010)	McCrae (2020)	Miller-Matero (2022)
Reporting																				
1	Is the hypothesis/aim/objective of the study clearly described? (1 = yes, 0 = no)	1	1	1	1	1	1	1	1	1	1	1	1	1	1	1	0	1	1	1
2	Are the main outcomes to be measured clearly described in the introduction or method section? (1 = yes, 0 = no)	1	1	1	1	1	1	1	1	1	0	1	1	1	1	1	1	1	1	1
3	Are the characteristics of the patients included in the study clearly described?	1	1	1	1	1	1	1	1	1	1	1	1	1	1	1	1	1	1	1

	(1 = yes, 0 = no)																			
4	Are the interventions of interest clearly described? (1 = yes, 0 = no)	1	1	1	1	1	1	1	1	1	0	1	1	1	1	1	1	1	1	1
5	Are the distributions of principal confounders in each group of subjects to be compared clearly described? (2 = yes, 1 = partially, 0 = no)	2	2	2	2	2	2	2	2	2	1	2	2	2	2	2	2	2	2	2
6	Are the main findings of the study clearly described? (1 = yes, 0 = no)	1	1	1	1	1	1	1	1	1	1	1	1	1	1	1	1	1	1	1
7	Does the study provide estimates of the random variability in the data for the main outcomes? (1 = yes, 0 = no)	1	1	1	1	1	1	1	1	1	0	1	1	1	1	1	1	0	1	1
8	Have all important adverse events that may be a consequence of the intervention been reported? (1 = yes, 0 = no)	1	1	1	1	1	1	1	1	1	1	1	1	1	1	1	1	1	1	1

9	Have the characteristics of patients lost to follow-up been described? (1 = yes, 0 = no)	1	1	1	1	1	1	1	1	1	1	1	1	1	1	1	1	1	1	1
10	Have the actual probability values been reported (e.g., 0.035 rather than <0.05) for the main outcomes except where the probability value is less than 0.001? (1 = yes, 0 = no)	1	1	1	1	1	0	0	1	1	0	0	1	1	0	1	0	0	1	1
Reporting Subtotal (score range, 0-11)		11	11	11	11	11	10	10	11	11	6	10	11	11	10	11	9	9	11	11
**Author First Name and Year**		Zeliadt (2022)	Huffman (2019)	Townsend (2008)	Ward (2022)	Van Der Merwe (2020)	Hooten (2007)	Davis (2018)	Schumann (2020)	Gibson (2020)	Van Hooff (2012)	Gilliam (2020)	Trinh (2023)	Passmore (2022)	Buchfuhrer (2023)	Barrett (2021)	Matyac (2022)	Nilsen (2010)	McCrae (2020)	Miller-Matero (2022)
External Validity																				
11	Were the subjects asked to participate in the study representative of the entire population from which they were recruited? (1 = yes, 0 = no)	1	1	1	1	1	1	1	1	1	1	1	1	1	1	1	1	0	0	1
12	Were those subjects who were prepared to	1	1	1	1	1	1	1	1	1	1	0	1	1	0	1	0	0	0	0

	participate representative of the entire population from which they were recruited? (1 = yes, 0 = no)																			
13	Were the staff, places, and facilities where the patients were treated representative of the treatment the majority of patients receive? (1 = yes, 0 = no)	1	1	1	1	1	1	1	1	1	1	1	1	1	1	1	1	1	1	1
External Validity Subtotal (score range, 0-3)		3	3	3	3	3	3	3	3	3	3	2	3	3	2	3	2	1	1	2
Internal Validity - Bias																				
14	Was an attempt made to blind study subjects to the intervention they have received? (1 = yes, 0 = no)	0	0	0	0	0	0	0	0	0	0	0	0	0	0	0	0	0	0	0
15	Was an attempt made to blind those measuring the main outcomes of the intervention?	0	0	0	0	0	0	0	0	0	0	0	0	0	0	0	0	0	0	0

	(1 = yes, 0 = no)																			
16	If any of the results of the study were based on "data dredging", was this made clear? (1 = yes, 0 = no)	1	1	1	1	1	1	1	1	1	1	1	1	1	1	1	1	1	1	1
17	In trials and cohort studies, do the analyses adjust for different lengths of follow-up of patients, or in case–control studies, is the time period between the intervention and outcome the same for cases and controls? (1 = yes, 0 = no)	1	0	1	1	1	1	1	1	0	1	1	1	1	1	1	1	1	1	1
18	Were the statistical tests used to assess the main outcomes appropriate? (1 = yes, 0 = no)	1	1	1	1	1	1	1	1	1	1	1	1	1	1	1	1	1	1	1
19	Was compliance with the intervention(s) reliable?	1	1	1	1	1	1	1	1	1	1	1	1	1	1	1	1	1	1	1

	(1 = yes, 0 = no)																			
20	Were the main outcome measures used accurate (valid and reliable)? (1 = yes, 0 = no)	1	1	1	1	1	1	1	1	1	0	1	1	1	1	1	1	1	1	1
Internal Validity - Bias Subtotal (score range, 0-7)		5	4	5	5	5	5	5	5	4	4	5	5	5	5	5	5	5	5	5
Internal Validity - Confounding																				
**Author First Name and Year**		Zeliadt (2022)	Huffman (2019)	Townsend (2008)	Ward (2022)	Van Der Merwe (2020)	Hooten (2007)	Davis (2018)	Schumann (2020)	Gibson (2020)	Van Hooff (2012)	Gilliam (2020)	Trinh (2023)	Passmore (2022)	Buchfuhrer (2023)	Barrett (2021)	Matyac (2022)	Nilsen (2010)	McCrae (2020)	Miller-Matero (2022)
21	Were the patients in different intervention groups (trials and cohort studies) or were the cases and controls (case–control studies) recruited from the same population? (1 = yes, 0 = no)	1	1	1	1	1	1	1	1	1	1	1	1	1	1	1	1	1	1	1
22	Were study subjects in different intervention groups (trials and cohort studies) or were the cases and controls (case–control studies) recruited over the	1	1	1	1	1	1	1	1	1	1	1	1	1	1	1	1	1	1	1

	same time period? (1 = yes, 0 = no)																			
23	Were the study subjects randomized to intervention groups? (1 = yes, 0 = no)	0	0	0	0	0	0	0	0	0	0	0	0	0	0	0	0	0	0	1
24	Was the randomized intervention assignment concealed from both patients and health care staff until recruitment was complete and irrevocable? (1 = yes, 0 = no)	0	0	0	0	0	0	0	0	0	0	0	0	0	0	0	0	0	0	0
25	Was there adequate adjustment for confounding in the analyses from which the main findings were drawn? (1 = yes, 0 = no)	1	1	1	1	1	1	1	1	1	1	1	1	1	1	1	1	1	1	1
26	Were losses of patients to follow-up taken into account? (1 = yes, 0 = no)	1	1	1	1	1	1	1	1	1	1	1	1	1	1	1	1	1	1	1
	Internal Validity -	4	4	4	4	4	4	4	4	4	4	4	4	4	4	4	4	4	4	5

	Confounding Subtotal (score range, 0-6)																			
Power																				
27	Did the study mention having conducted a power analysis to determine the sample size needed to detect a significant difference in effect size for one or more outcome measure? (2 = yes, ≥2 outcomes, 1 = yes, 1 outcome, 0 = no)	0	1	0	0	0	0	1	0	0	0	0	0	0	0	0	0	0	0	0
Power Subtotal (score range, 0-2)		0	1	0	0	0	0	1	0	0	0	0	0	0	0	0	0	0	0	0
Total Quality Index Score (range, 0-29 points)		23	23	23	23	23	22	23	23	22	17	21	23	23	21	23	20	19	21	23
